# Mass spectrometry and machine learning for the accurate diagnosis of benzylpenicillin and multidrug resistance of *Staphylococcus aureus* in bovine mastitis

**DOI:** 10.1371/journal.pcbi.1009108

**Published:** 2021-06-11

**Authors:** Necati Esener, Alexandre Maciel-Guerra, Katharina Giebel, Daniel Lea, Martin J. Green, Andrew J. Bradley, Tania Dottorini

**Affiliations:** 1 University of Nottingham, School of Veterinary Medicine and Science, Sutton Bonington, United Kingdom; 2 University of Nottingham School of Computer Science, Jubilee Campus, Nottingham, United Kingdom; 3 Quality Milk Management Services ltd, Easton, United Kingdom; 4 Digital Research Service, University of Nottingham, Sutton Bonington, United Kingdom; University of Cambridge, UNITED KINGDOM

## Abstract

*Staphylococcus aureus* is a serious human and animal pathogen threat exhibiting extraordinary capacity for acquiring new antibiotic resistance traits in the pathogen population worldwide.

The development of fast, affordable and effective diagnostic solutions capable of discriminating between antibiotic-resistant and susceptible *S*. *aureus* strains would be of huge benefit for effective disease detection and treatment. Here we develop a diagnostics solution that uses Matrix-Assisted Laser Desorption/Ionisation–Time of Flight Mass Spectrometry (MALDI-TOF) and machine learning, to identify signature profiles of antibiotic resistance to either multidrug or benzylpenicillin in *S*. *aureus* isolates. Using ten different supervised learning techniques, we have analysed a set of 82 *S*. *aureus* isolates collected from 67 cows diagnosed with bovine mastitis across 24 farms. For the multidrug phenotyping analysis, LDA, linear SVM, RBF SVM, logistic regression, naïve Bayes, MLP neural network and QDA had Cohen’s kappa values over 85.00%. For the benzylpenicillin phenotyping analysis, RBF SVM, MLP neural network, naïve Bayes, logistic regression, linear SVM, QDA, LDA, and random forests had Cohen’s kappa values over 85.00%. For the benzylpenicillin the diagnostic systems achieved up to (mean result ± standard deviation over 30 runs on the test set): accuracy = 97.54% ± 1.91%, sensitivity = 99.93% ± 0.25%, specificity = 95.04% ± 3.83%, and Cohen’s kappa = 95.04% ± 3.83%. Moreover, the diagnostic platform complemented by a protein-protein network and 3D structural protein information framework allowed the identification of five molecular determinants underlying the susceptible and resistant profiles. Four proteins were able to classify multidrug-resistant and susceptible strains with 96.81% ± 0.43% accuracy. Five proteins, including the previous four, were able to classify benzylpenicillin resistant and susceptible strains with 97.54% ± 1.91% accuracy. Our approach may open up new avenues for the development of a fast, affordable and effective day-to-day diagnostic solution, which would offer new opportunities for targeting resistant bacteria.

## Author summary

Antibiotic resistance is one of the biggest threats to human and animal health. The incessant emergence of new multidrug-resistant bacteria needs to be counterbalanced by the implementation of effective diagnostics solutions to detect resistance and support treatment selection. The objective of this study is the development of effective diagnostic solutions to identify resistance to benzylpenicillin and other drugs in *S*. *aureus* strains infecting dairy cattle. *S*. *aureus* is one of the most common pathogens of clinical mastitis in the dairy industry, affecting productivity, profitability, animal health and welfare, and has an extraordinary capacity for acquiring new antibiotic resistance traits. Our diagnostic solution combines machine learning and mass spectrometry. The application to a test set of 82 *S*. *aureus* isolates collected from 67 cows diagnosed with bovine mastitis across 24 farms discriminated between multidrug-resistant and susceptible strains with (mean result ± standard deviation over 30 runs on the test set) 96.81% ±0.43% accuracy, and between benzylpenicillin-resistant and susceptible strains with 97.54% ± 1.91% accuracy. Through a dedicated bioinformatics pipeline developed on the results of machine learning, we were able to obtain new insights into the molecular determinants and mechanism underlying the antibiotic resistance phenotypes. We believe that our approach may open up new avenues for the development of a fast, affordable and effective diagnostic solution which would offer new opportunities for targeting resistant bacteria and support with timely, accurate and targeted treatment selection.

## Introduction

*Staphylococcus aureus* is a major opportunistic pathogen, infecting both humans and a wide variety of animals including dairy cattle, which have been recently proven to pose an important zoonotic potential, being the principal animal reservoir of novel human epidemic clones [1]. Worldwide, *S*. *aureus* is one of the most frequently isolated pathogens of bovine mastitis, which remains a significant problem in the dairy industry by affecting productivity, profitability, animal health and welfare [2]. The majority of bovine mastitis infections caused by *S*. *aureus* exhibit subclinical and chronic manifestations resulting in long-term intramammary persistence [3]. *S*. *aureus* can reproduce swiftly upon entering the mammary gland and induce immune reactions that can lead to tissue injuries [4]. Most of the time, the immune response of the cow itself cannot successfully eliminate the *S*. *aureus* infection and treatment is needed [4]. Existing *S*. *aureus* vaccines are not considered as a preventive solution due to their yet unproven effectiveness against infections [5].

In 2000, Gentillini *et al*. [6] indicated that beta-lactams (penicillins and cephalosporins), aminoglycosides, macrolides and lincosamides were the most commonly used antibiotics for treatment of bovine mastitis. In addition, according to a recent survey [7] in 2018, penicillins, aminoglycosides and third/fourth generation cephalosporins were the most common antibiotics used on the treatment for bovine mastitis in the UK. The first examples of using benzylpenicillin for bovine mastitis treatment can be traced back to the 1940s [8]. However, penicillin-resistant *S*. *aureus* strains, carrying a penicillinase/beta-lactamase emerged shortly after its first clinical usage and by the early 1950s, they became pandemic [8]. In 1959 a penicillin derivative, methicillin, that was resistant to β-lactamase hydrolysis was synthetized. However, immediately after methicillin was used clinically, methicillin-resistant *S*. *aureus* (MRSA) strains were isolated [9,10]. Resistance to methicillin is conferred by the acquisition of a mobile genetic element, the staphylococcal cassette chromosome (SCCmec) carrying the gene *mecA* encoding a penicillin-binding protein (PBP2a) [9,10]. Over the years, mutations, acquisition and accumulation of antibiotic resistance-conferring genes, divergent *mecA* gene homologues (*mecC*) [11,12] and SCCmec elements [11] have led to the emergence of multi-resistant MRSA strains [13].

Nowadays, MRSA are resistant to virtually all β-lactam antibiotics [11]. Since its emergence in the early 2000’s, livestock-associated methicillin-resistant *S*. *aureus* (LA-MRSA) has become an emerging problem in many parts of the world [14–16]. The detection of *mecC* MRSA from dairy cattle in England [12] was reported in 2011. The first isolation of both *mecA* and *mecC* LA-MRSA. In bulk milk from dairy cattle in the UK was reported in 2012 [17]. Worryingly, a number of studies have suggested possible human-livestock MRSA transmissions [16,18–20]. In addition, several studies have reported that persons with occupational livestock exposure may be at increased risk of becoming colonized with LA-MRSA [21]. More than 90% of current human-associated isolates [22] and varying from 84% to 92% of dairy-related isolates were observed to be penicillin-resistant [23, 24]. However, the UK surveillance report between 2016 and 2018 showed that penicillin resistance in *S*. *aureus* was relatively low (20.4% on average) in British dairy cattle [25].

It is not uncommon in dairy cattle practice to give antibiotics to healthy animals to prevent the insurgence of diseases, and to sick animals often without certainty about the actual bacterial origin of the disease. Even when the disease is of recognised bacterial origin, broad-spectrum antibiotics are often used, instead of targeting the specific bacterial strain causing the illness. Underlying such prescription practices is the lack of fast, affordable and effective diagnostic solutions, which leaves the veterinarian to primarily rely on educated guesses. These practices have serious consequences, amongst which is the appearance and diffusion of multidrug antibiotic resistance profiles in the pathogen population.

*S*. *aureus* has an extraordinary capacity of acquiring new resistance traits by the integration into its genome of exogenous genetic material via horizontal gene transfer and mutational events [26,27]. In *Staphylococcus* spp, the major targets underlying mechanisms of resistance are the cell envelope, the ribosome and nucleic acids [28]. However, several studies have identified hypothetical proteins as also being associated with drug resistance specifically in *S*. *aureus* [29].

Characterising the proteins, alone or in combination, that contribute to resistance, can potentially lead to improved diagnostic tools and therapeutics against antibiotic-resistant *S*. *aureus* and may hold the key to unlocking this global health problem. In veterinary medicine, the identification of multidrug-resistant (MDR) pathogens and the identification of their antibiotic resistance profiles is done by conventional methods such as disk diffusion, epsilometer test, Vitek, macrodilution and microdilution [30]. However, such diagnostic tools are not affordable and quick enough to offer real-time control of invasive infections.

Matrix-Assisted Laser Desorption/Ionisation–Time of Flight Mass Spectrometry (MALDI-TOF) has been an alternative way of detecting antibiotic resistance thanks to its low-cost and speed [31]. Antibiotic resistance profiles of several organisms could be determined by MALDI-TOF [32–34], and, in combination with machine learning techniques, larger datasets, a wide range of microbial species identification and complex antimicrobial resistance profile could be analysed faster and more easily and economically, revolutionizing the field of microbiology [35]. *S*. *aureus* was one of the most frequently studied genera for antimicrobial resistance prediction [36–40]. Rapid and accurate classification of MRSA and methicillin-sensitive *S*. *aureus* (MSSA) based on MALDI-TOF spectral of clinical samples were obtained by several studies [36,38,39]. Analogously, high accuracy results have been obtained when applying machine learning approaches to MALDI-TOF spectral data for the prediction of the broad-spectrum antibiotic vancomycin. In particular, successful separation of vancomycin-intermediate (VISA) from vancomycin-susceptible *S*. *aureus* (VSSA) on the basis of MALDI-TOF data collected from clinical samples [37,40,41]. Recently, van Oosten and Klein [42], developed classification models for *S*. *aureus* which assign the mechanisms of action of antibacterial drugs.

The objective of this study was to find a fast and more accurate alternative to standard susceptibility tests, to profile multidrug and benzylpenicillin resistance in *S*. *aureus* isolates. To this end, we tested the discriminatory power given by the combination of supervised machine learning and MALDI-TOF, complemented by a protein-protein interaction (PPI) network and a protein structural analysis workflow. Here for the first time, we demonstrate that this approach can be used to develop diagnostic solutions that can discriminate with high performance between benzylpenicillin- and multidrug-resistant and susceptible bovine mastitis-causing *S*. *aureus* isolates.

## Results

### Sample analysis

**Fig 1:**
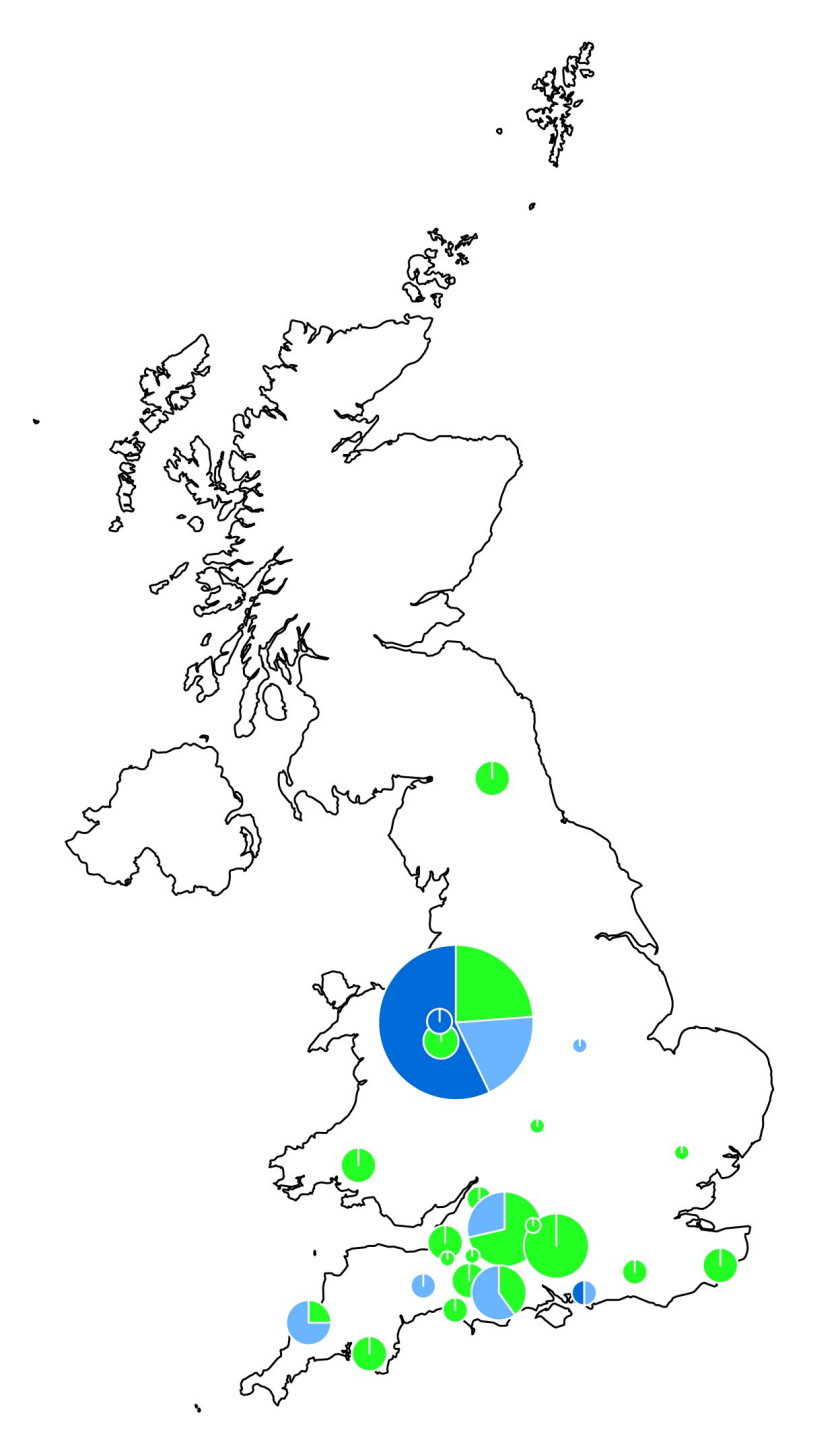
Location of the enrolled farms in the United Kingdom. The circles represent the location of the farms and the size of the circles indicate the number of *S*. *aureus* isolates in the farms. The highest number of isolates provided by a single farm was 21, while the lowest was 1. The green colour represents the susceptible *S*. *aureus* isolates while the dark and light blue is for multidrug-resistant and benzylpenicillin-resistant only *S*. *aureus* isolates, respectively. Natural Earth was used as base to construct the map for the United Kingdom (https://www.naturalearthdata.com/downloads/10m-cultural-vectors/10m-admin-0-countries/), the map was created with rnaturalearth package in R.

In this study, 82 *S*. *aureus* isolates had been cultured from milk samples collected between March 2004 and May 2005. The samples were from 24 herds each in a different farm (24 farms) where 23 farms were in England (most of them in the south) and one farm was in Wales (Llangathen, Carmarthen). The locations of the farms and *S*. *aureus* isolates collected from each farm are shown in Fig 1 and the breakdown of isolates per farm is shown on S1 Table. Moreover, S2 Table indicates the antimicrobial susceptibility profile of the resistant isolates that were obtained from the same animal.

**Fig 2:**
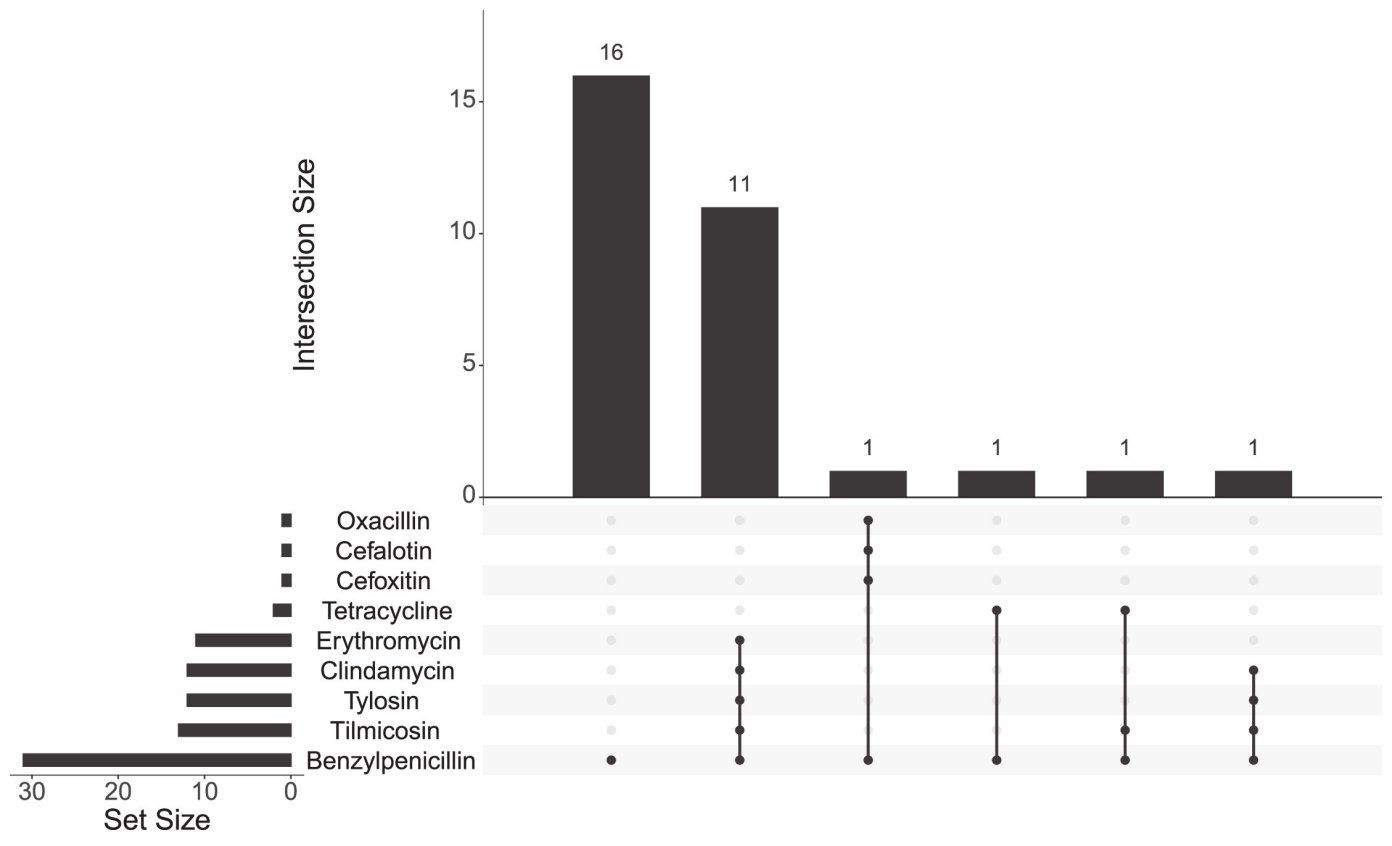
UpSet plot comparing the profiles of benzylpenicillin-resistant *Staphylococcus aureus* isolates. The total size of resistant *S*. *aureus* isolates is shown on the left bar plot. Antibiotic-resistant profiles of *S*. *aureus* isolates are visualized by the bottom plot and the occurrence is represented on the top bar plot.

VITEK analysis showed that the cohort consisted of 31 benzylpenicillin-resistant and 51 benzylpenicillin-susceptible isolates. Amongst the resistant isolates, 16 isolates were found to be only penicillin-resistant, while 15 isolates had resistance to multiple antibiotics, among these 15 isolates 13 were found to be resistant to three or more antibiotics, with at least one antimicrobial agent in three antimicrobial classes (multidrug-resistant, MDR), while two isolates were resistant to two or more antibiotics with at least one antimicrobial agent in two antimicrobial classes (extensively drug-resistant, XDR). We considered the MDR and XDR as one class and named it as MDR for simplicity. As shown in Fig 2, out of 15 multidrug-resistant isolates, 11 isolates were resistant to benzylpenicillin, clindamycin, erythromycin, tilmicosin and tylosin; 1 isolate was resistant to benzylpenicillin, clindamycin, tilmicosin and tylosin; 1 isolate was resistant to benzylpenicillin, tetracycline and tilmicosin; 1 isolate was resistant to benzylpenicillin and tetracycline, and 1 isolate was resistant to benzylpenicillin, cefalotin, cefoxitin and oxacillin. 51 isolates were found to be susceptible to all antibiotics used in this study which were benzylpenicillin, cefoxitin, oxacillin, cefalotin, ceftiofur, cefquinome, amikacin, gentamicin, kanamycin, neomycin, enrofloxacin, clindamycin, erythromycin, tilmicosin, tylosin, tetracycline, florfenicol and trimethoprim/sulfamethoxazole.

### Generation of MALDI-TOF peak lists and set-up of the classifiers

A total of 312 MALDI-TOF raw data spectra had been obtained from 82 *S*. *aureus* isolates, on average 4 replicate spectra per isolate. The peak lists, i.e. the lists of paired mass/charge (*m/z*) ratios and corresponding intensity values, were extracted from the raw spectra as specified in the Methods Section.

Supervised machine learning algorithms were used to implement classifiers to verify if the MALDI-TOF peaks associated with isolates could be used to predict their resistance or susceptibility to benzylpenicillin and multidrug. Being based on supervised learning, all methods required the availability of training datasets for model building and validation datasets for assessing the performance of the classifier. The prediction performance of each classifier was evaluated measuring accuracy, sensitivity, specificity and kappa. Thirty iterations of nested cross-validation (described in Methods) were used to train each classifier.

The following classification methods, available in the scikit-learn library in Python, were tested: naïve Bayes, linear and non-linear (RBF kernel) support vector machines (SVM), decision tree, random forests, multi-layer perceptron neural networks (MLP), AdaBoost (AdaBoost-SAMME version), logistic regression, linear discriminant analysis (LDA) and quadratic discriminant analysis (QDA).

### Analysis of multidrug-resistant vs susceptible isolates

**Fig 3:**
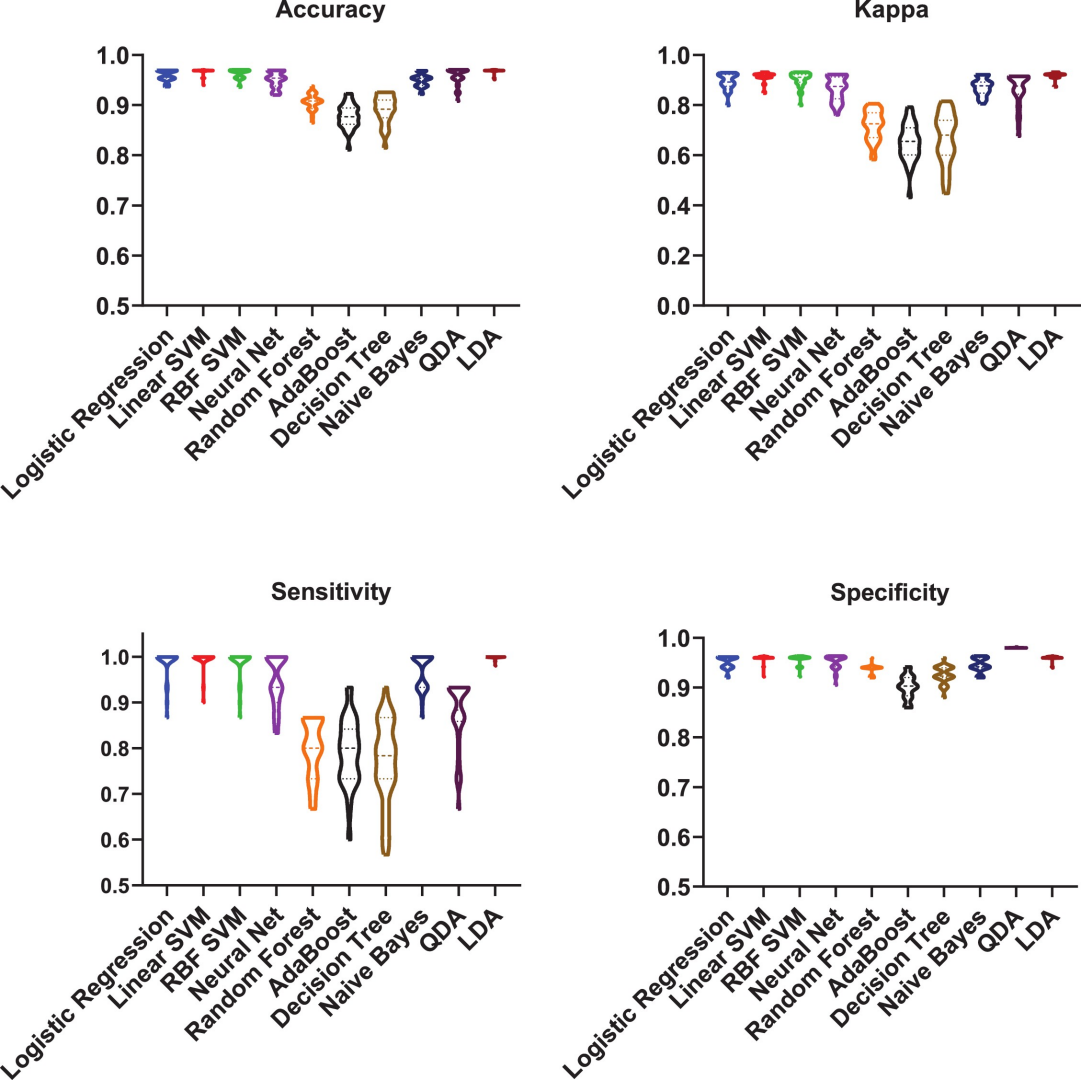
Supervised machine learning prediction of multidrug resistance spectral signature profiles. Prediction performance results of different classifiers (logistic regression, linear SVM, RBF SVM, MLP neural network, decision tree, random forest, AdaBoost, naïve Bayes, quadratic discriminant analysis (QDA) and linear discriminant analysis (LDA)) that were used to classify the multidrug resistance profiles are shown on the X-axis. Four performance indicators have been used to evaluate the classification: accuracy, kappa, sensitivity and specificity. The scores for each performance metric are indicated in the Y-axis.

We first focused on investigating the possibility to develop a classifier to verify if MALDI-TOF peak lists associated with isolates could be used to predict their multidrug phenotype. Specifically, we considered the spectra of 15 multidrug-resistant isolates (13 MDR and 2 XDR) and 51 susceptible isolates (susceptible to all antibiotics tested in this study). A total of 249 raw spectra were analysed. The pre-processing led to the identification of four different peaks (Table 1) found to appear in at least 30% of all number of spectra. Due to the unbalanced nature of this specific data set (76% of samples were susceptible and only 24% were resistant), we first standardised the four features by a down-sampling method to build robust classifiers [43]. At each one of the 30 runs, 15 samples were randomly chosen out of the initial 51 susceptible samples and a final balanced (50% resistant, 50% susceptible) dataset was generated. The four peaks were then used as features to build ten classifiers and to develop predictive models for the multidrug phenotype. Before the classification, features were standardised (mean centred and unit variance) then resistant and susceptible isolates were labelled as 0 and 1, respectively. 30 runs using nested cross-validation were performed. Amongst the investigated machine learning approaches, LDA, linear SVM and RBF SVM were found as the top three best performance showing algorithms, respectively. Diagnostic systems trained on individual isolates coming from 24 different farms achieved up to (mean result ± standard deviation over 30 runs on the test set): accuracy = 96.81% ±0.43%, sensitivity = 99.88% ± 0.41%, specificity = 95.96% ± 0.52%, and kappa = 91.83% ± 1.37% in LDA algorithm. Detailed performance results of all classifiers on test data can be found in Fig 3.

**Table 1:** Statistical evaluation of the 4 peaks with an overall frequency of appearance higher than 30% based on the multidrug resistant vs susceptible data set.

Mass (kDa)	PTTA	PWKW	Ave1	Ave2	StdDev1	StdDev2	PA	PA1	PA2
4.807	3.78E-12	1.34E-07	7.27	19.55	5.89	3.72	66.88	35.71	98.04
6.422	0.00036	0.041891	6.92	10.30	4.54	2.00	45.31	35.71	54.90
6.891	0.02021	0.12752	31.98	43.04	23.96	14.89	80.18	64.29	96.07
9.621	6.81E-08	3.73E-07	32.39	43.00	3.28	6.23	100.00	100.00	100.00

**PTTA** is the *p*-value of Welch‘s *t*-test; **PKWK** is the *p*-value of Wilcoxon test; index 1 refers to resistant isolates; index 2 refers to susceptible isolates; **Ave** is the overall intensity average; **Ave1** is the intensity average of class ‘Resistant’; **Ave2** is the intensity average of class ‘Susceptible’; **StdDev** is the overall intensity standard deviation; **StdDev1** is the intensity standard deviation of class ‘Resistant’; **StdDev2** is the intensity standard deviation of class ‘Susceptible’; **PA** is the overall proportion of appearance; **PA1** is the proportion of appearance of class ‘Resistant’; **PA2** is the proportion of appearance of class ‘Susceptible’.

### Analysis of benzylpenicillin-resistant only vs susceptible isolates

Next, we investigated resistance and susceptibility to benzylpenicillin only. This was to isolate specific patterns underlying resistance to this specific antibiotic. We chose benzylpenicillin because it was the only antibiotic for which we had singly resistant isolates.

**Fig 4:**
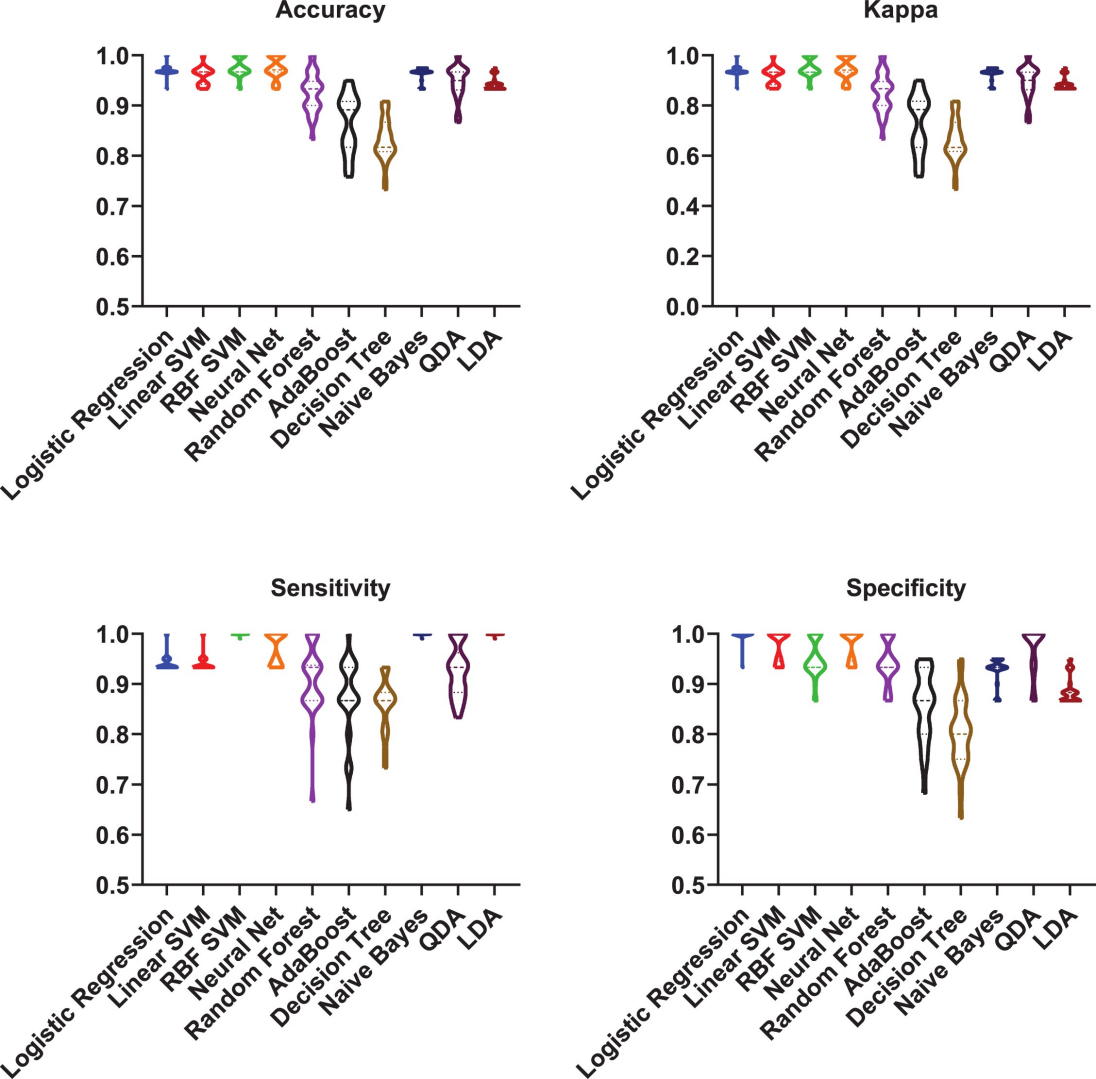
Supervised machine learning prediction of benzylpenicillin resistance spectral signature profiles. **Prediction performance results of** ten different classifiers (logistic regression, linear SVM, RBF SVM, MLP neural network, decision tree, random forest, AdaBoost, naïve Bayes, quadratic discriminant analysis (QDA) and linear discriminant analysis (LDA)) that were used to classify the benzylpenicillin resistance profiles are shown on the X-axis. Four performance indicators have been used to evaluate the classification: accuracy, kappa, sensitivity and specificity. The scores for each performance metric are indicated in the Y-axis.

To this aim, the spectra of the 16 benzylpenicillin-resistant only isolates and 51 susceptible isolates (susceptible to all antibiotics tested in this study) were first pre-processed as described in the Methods Section. Five peaks were found in at least 30% of the overall number of spectra (Table 2). Due to the unbalanced nature of this specific data set (76% of samples are susceptible and only 24% are resistant), we first standardised the five features by a down-sampling method to build robust classifiers [43]. At each one of the 30 runs, 16 samples were randomly chosen out of the initial 51 susceptible samples and a final balanced (50% resistant, 50% susceptible) dataset was generated. The five peaks were then used as features to build ten classifiers and to develop predictive models for the benzylpenicillin phenotype. Before the classification, features were standardised (mean centred and unit variance) then resistant and susceptible isolates were labelled as 0 and 1, respectively. 30 runs using nested cross-validation was performed. Amongst the investigated machine learning approaches RBF SVM, neural network and logistic regression were those that achieved the best performance. Diagnostic systems trained on individual isolates coming from 24 different farms achieved up to (mean result ± standard deviation over 30 runs on the test set); accuracy = 97.54% ± 1.91%, sensitivity = 99.93% ± 0.25%, specificity = 95.04% ± 3.83%, and kappa = 95.04% ± 3.83% in RBF SVM algorithm. Detailed performance results of all classifiers on test data can be found in Fig 4.

**Table 2:** Statistical evaluation of the 5 peaks with an overall frequency of appearance higher than 30% based on the benzylpenicillin resistant only vs susceptible data set.

Mass (kDa)	PTTA	PWKW	Ave1	Ave2	StdDev1	StdDev2	PA	PA1	PA2
4.305	0.258564	0.213998	10.20	9.34	2.60	2.64	34.33	37.50	33.33
4.807	7.02E-08	5.96E-07	12.94	19.55	4.02	3.72	92.54	75.00	98.04
6.422	0.39999	0.50342	10.81	10.30	2.44	2.00	58.21	68.75	54.90
6.891	5.69E-12	8.31E-08	10.00	43.04	8.80	14.89	76.12	56.16	96.07
9.621	1.81E-10	3.35E-08	29.84	43.00	5.54	6.23	100.00	100.00	100.00

**PTTA** is the *p*-value of Welch‘s *t*-test; **PKWK** is the *p*-value of Wilcoxon test; index 1 refers to resistant isolates; index 2 refers to susceptible isolates; **Ave** is the overall intensity average; **Ave1** is the intensity average of class ‘Resistant’; **Ave2** is the intensity average of class ‘Susceptible’; **StdDev** is the overall intensity standard deviation; **StdDev1** is the intensity standard deviation of class ‘Resistant’; **StdDev2** is the intensity standard deviation of class ‘Susceptible’; **PA** is the overall proportion of appearance; **PA1** is the proportion of appearance of class ‘Resistant’; **PA2** is the proportion of appearance of class ‘Susceptible’.

Notably, four peaks (4.807kDa, 6.422kDa, 6.891kDa and 9.621kDa) were found common in the analysis of benzylpenicillin-resistant vs susceptible and multidrug-resistant vs susceptible isolates. When comparing the intensities of these four peaks in the two datasets (resistant vs. susceptible) we observed that 4.807kDa, 6.891kDa and 9.621kDa had a higher average in susceptible isolates consistently while 6.422kDa had a higher average of intensity in benzylpenicillin-resistant only isolates class. 4.305kDa which was specific to benzylpenicillin-resistant only analysis had higher average intensity in resistant than susceptible isolates.

### Machine learning analyses undertaken to prove the effectiveness of our method to differentiate susceptibility/resistance profiles rather than strain differences

Because two of the five discriminant proteins found in this work were of ribosomal origins and ribosomal proteins have been used for the discrimination of major *S*. *aureus* lineages based on MALDI-TOF analysis [44–47], we performed further analyses in support that our classifiers were picking up susceptibility/resistance differences rather than strain differences. First, we investigated if and how in the sole presence of the ribosomal peaks as input features or in their absence the performance of the classifiers changed and how. As shown in S3 Table by removing only the ribosomal proteins from the analysis of both multidrug and benzyl-penicillin datasets, the performance of the classifiers decreases but not significantly, all indicators are still above 80%. However, when using only the ribosomal proteins as input features for the analysis of both multidrug and benzyl-penicillin datasets, the specificity and Cohen’s kappa indicators drop to unacceptable values for both the multidrug and benzyl-penicillin predicted phenotypes. Altogether these results indicate that the ribosomal proteins in combination with the other discriminant proteins are contributing to the susceptibility/resistance classification but do not play a major role in the classification.

### Biomarker characterization–identification of the proteins found to correspond to the MALDI-TOF spectral peaks recognised as discriminant by the trained classifiers

**Fig 5:**
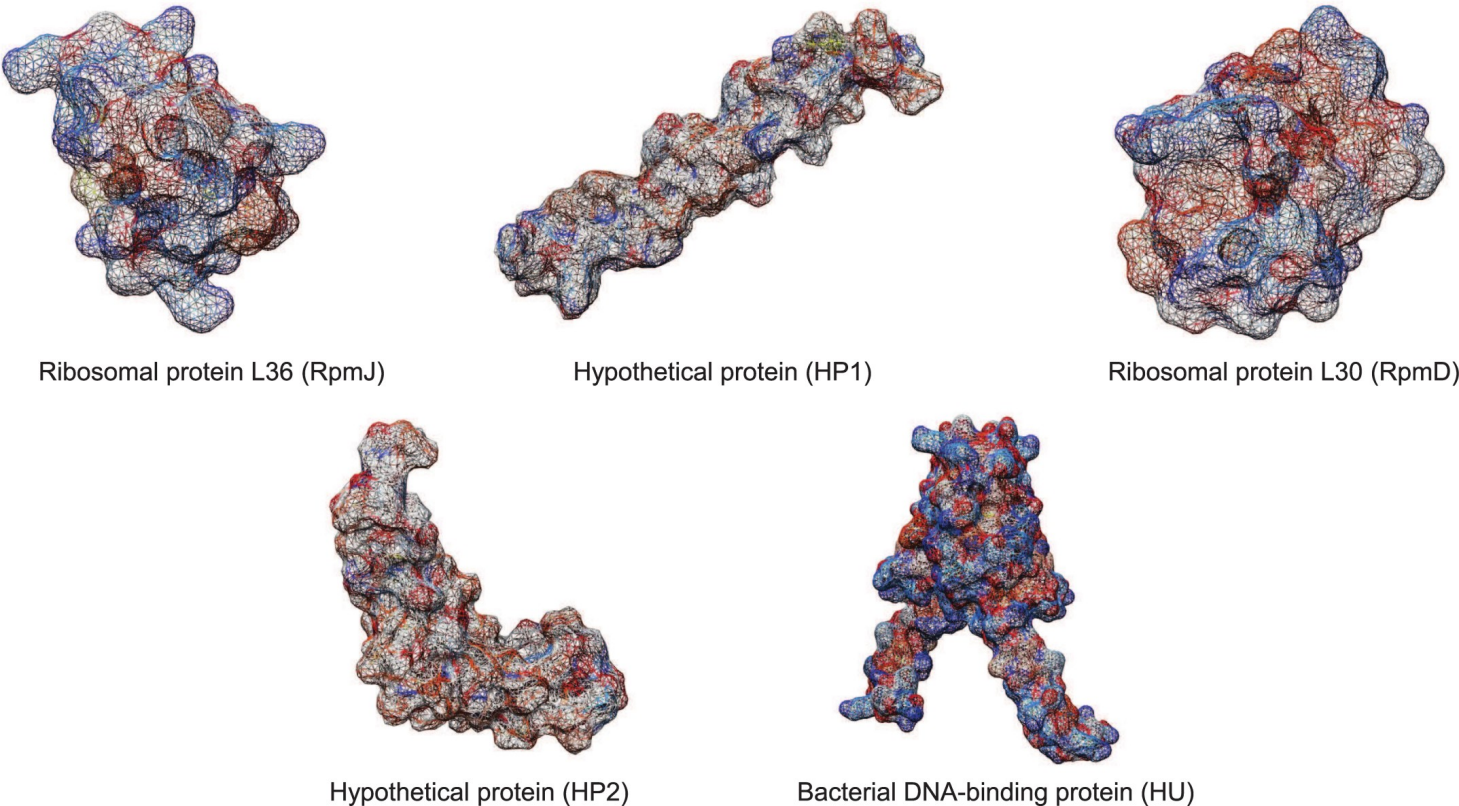
3D structures of the five proteins found to correspond to the MALDI-TOF spectral peaks recognized as discriminant between benzylpenicillin resistant and susceptible isolates. Top row from left to right: homology model of ribosomal protein L36p (RpmJ, mw: 4305.36Da), threading model of hypothetical protein (HP1, mw: 4801.95Da) and homology model of ribosomal protein L30p (RpmD, mw: 6422.48Da). Bottom row from left to right: threading model of hypothetical protein (HP2, mw: 6901.37Da) and homology model of bacterial DNA-binding protein (HU, mw: 9626.01Da).

The five peaks identified as providing optimal discrimination between benzylpenicillin-resistant only and susceptible isolates were further analysed to identify their correspondent *S*. *aureus* proteins. It should be noted that the four peaks identified as providing optimal discrimination between multidrug-resistant and susceptible were also amongst these peaks. When compared to the reference *S*. *aureus* Newbould 305 (ATCC 29740) proteome, the five peak masses identified the following five *S*. *aureus* proteins: two hypothetical proteins (molecular weights of 4801.95 and 6901.37 Da), RpmJ, RpmD and DNA-binding protein HU. The molecular weights of the corresponding proteins changed slightly from those in the original spectra as a result of the search criteria outlined in the Methods (Table 3). In order to better understand the functions and roles of these proteins within the drug resistance phenotype, we characterised the molecular functions (MF), cellular components (CC), and biological processes (BP) they may carry out. RpmJ and RpmD are the 50S ribosomal proteins L36 and L30, respectively. HU is a histone-like DNA-binding protein, which interacts with DNA to protect from denaturation [48]. For the hypothetical proteins, we used 3D threading methods to predict the Gene Ontology (GO) functions (Fig 5). The hypothetical protein of 4801.95Da was annotated as COPII-coated vesicle cargo loading (BP), intracellular protein transport (BP), proteolysis (BP), homophilic cell adhesion via plasma membrane adhesion molecules (BP) and ion binding (MF). The hypothetical protein of 6901.37Da was annotated as being involved with the small molecule metabolic process (BP), antibiotic metabolic process (BP), lipid transport (BP) and ion binding (MF).

**Table 3:** Annotation of the *S*. *aureus* proteins corresponding to the five MALDI-TOF peaks recognized as significant by the trained classifiers: peak mass charge ratio, predictedprotein mass, top PSI-BLAST matches, conserved domain analyses, cellular locations and overexpressed classes are shown.

MALDI-TOF Peak	Protein (MW)	PSI-BLAST Match	Identity (e-value)	Domain (e-value)	PSORTB location (score)	Overexpressed Class
*m/z* 4305.59	RpmJ (4305.36Da)	50S ribosomal protein L36	100.00% (4e-16)	Ribosomal_L36 (1.2e-19)	Cytoplasmic (10.00)	Benzylpenicillin resistant isolates
*m/z* 4807.21	HP1 (4801.95Da)	Uncharacterized protein	100.00% (4e-14)	No conserved domain was identified.	Cytoplasmic membrane (9.55)	Susceptible isolates
*m/z* 6422.37	RpmD (6422.48Da)	50S ribosomal protein L30	100.00% (4e-33)	Ribosomal_L30 (3.4e-21)	Cytoplasmic (9.67)	Benzylpenicillin resistant isolates
*m/z* 6891.17	HP2 (6901.37Da)	Membrane protein	100.00% (1e-07)	No conserved domain was identified.	Cytoplasmic membrane (9.55)	Susceptible isolates
*m/z* 9621.26	DNA-binding protein HBsu (9626.01Da)	HU family DNA-binding protein	100.00% (2e-56)	Bacterial DNA-binding protein (6.2e-37)	Cytoplasmic (9.67)	Susceptible isolates

HP: hypothetical protein. Column 1 shows the mass charge ratio of the MALDI-TOF peaks identified by the machine learning framework; column 2 shows the predicted molecular weights of the proteins corresponding to the MALDI-TOF peaks; column 3 shows best PSI-BLAST matches; column 4 shows the identities and e-values obtained with the PSI-BLAST matches; column 5 shows the domain and e-value predicted with CDD database; column 6 shows the results obtained with the PSORTB predictor; and column 7 shows the overexpressed class where the corresponding proteins have the highest intensity.

With the aim to further characterise the function of these proteins we did a PSI-BLAST comparative analysis; all discriminant proteins with 100% coverage and significant e-values are shown in Table 3.

**Fig 6:**
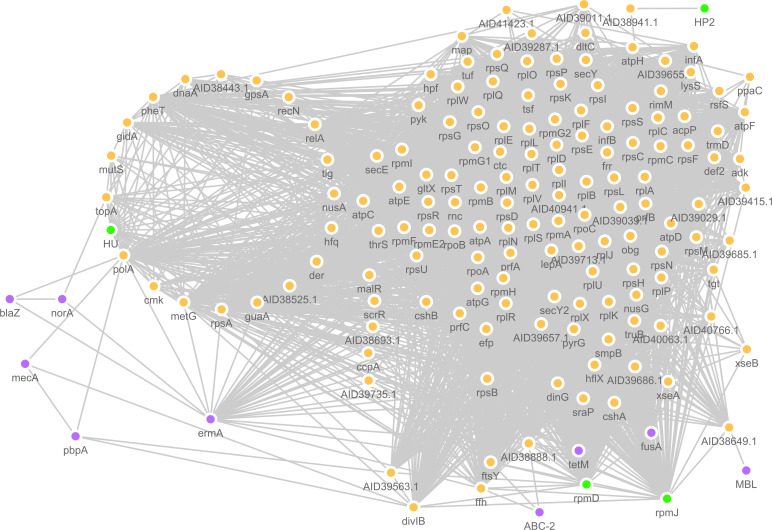
Protein-protein interaction network of the proteins found to correspond to the MALDI-TOF spectral peaks recognized as discriminant between benzylpenicillin resistant and susceptible isolates. The PPI network showing the four discriminant proteins, green circles, (RpmJ, RpmD, HU and hypothetical protein 2 (HP2)) and their first neighbour interactors (orange colours). Amongst these first shell interacting partners, purple nodes represent the antibiotic-resistant proteins (BlaZ, NorA, MecA, PbpA, ErmA, ABC-2, TetM, FusA and MBL) predicted by ResFinder v3.1 [94].

**Fig 7:**
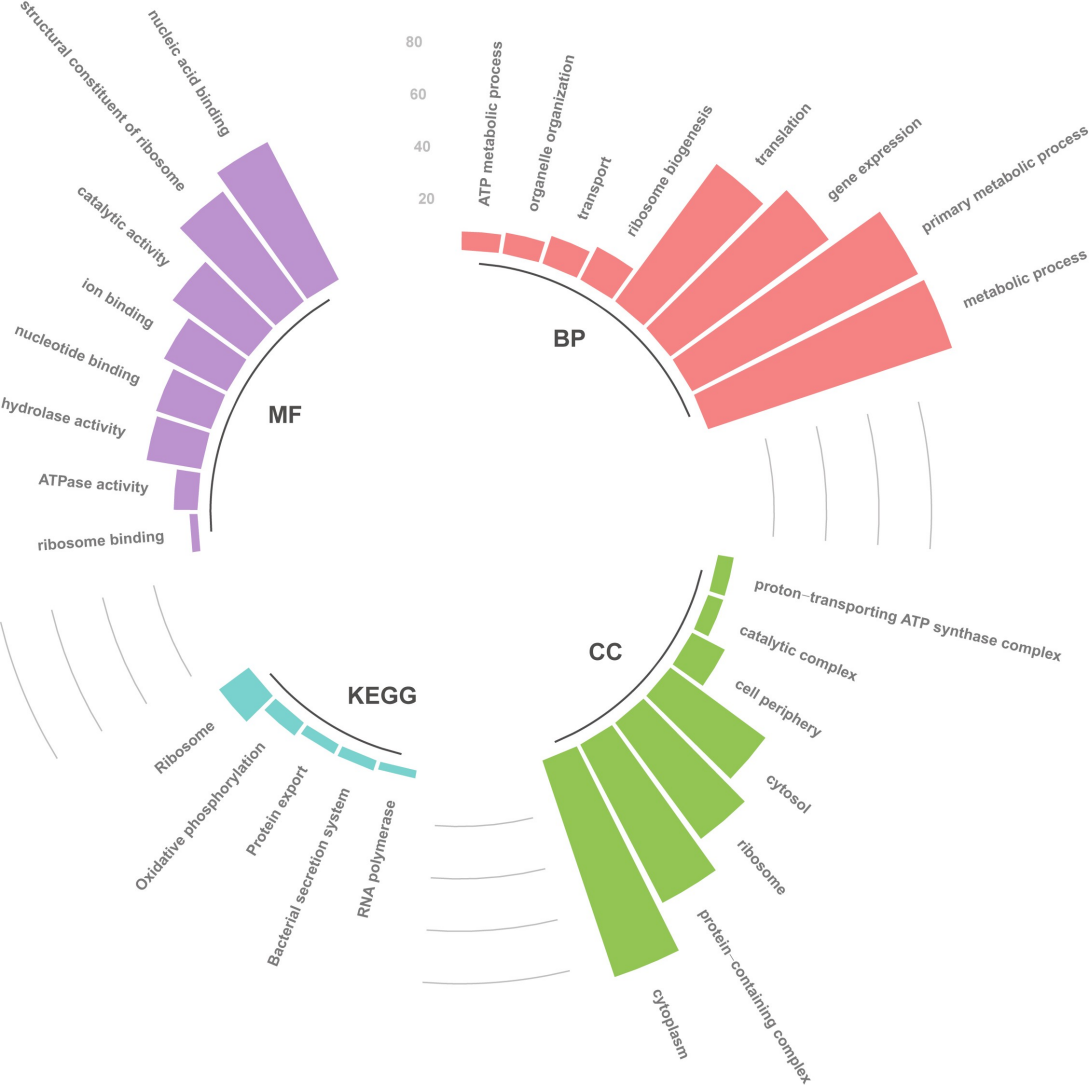
Functional enrichment analysis of the benzylpenicillin network in *Staphylococcus aureus* based on Gene Ontology (GO) and Kyoto Encyclopedia of Genes and Genomes (KEGG) pathways. The network contains the 4 discriminant proteins, that were found to be discriminant between benzylpenicillin resistant and susceptible isolates, and their 149 first neighbours. GO consists of cellular component (CC), molecular function (MF) and biological process (BP). In each ontology, the enriched categories and the number of genes populating them are shown. Likewise, the enriched KEGG pathways and the number of genes populating each pathway are indicated.

Next, we investigated the drug resistance interactome by building the protein-protein interaction network. The benzylpenicillin PPI network, including the four significant proteins (RpmJ, RpmD, HU and HP2) and their 149 first neighbours, was generated (Fig 6). It should be noted that HP1 could not be found in the *S*. *aureus* proteome that was available in STRING database. GO and KEGG analyses of the network showed enrichment for ribosome, nucleic acid binding and catalytic activity (Fig 7).

Tetracycline resistance protein (TetM) and elongation factor G (FusA) were found as the first neighbours of RpmJ and RpmD based on the experimental findings of their homologs in *E*. *coli* [49, 50]. Additional four proteins (MecA, BlaZ, PbpA and metallo-beta-lactamase (MBL)) were associated with beta-lactams, rRNA adenine N-6-methyltransferase (ErmA), macrolides resistance, multidrug efflux pump (NorA) and ABC transporter protein (ABC-2). These proteins were found to interact with some first neighbours of the discriminant proteins in the network. Penicillin-binding protein 2 prime (MecA) was shown to share a common interactor, cell division protein (DivIB), with the discriminant protein RpmD. The interactions of MecA-DivIB (interaction score: 0.639) and DivIB-RpmD (interaction score: 0.864) are based on experimental/biological data coming from homologs in other species [51]. MecA was also shown to share a common interactor, DNA polymerase I (PolA), with the discriminant protein HU. While the interaction of MecA-PolA was based on text mining (interaction score: 0.432), the interaction of PolA-HU was based on experimental/biological data (interaction score: 0.668) obtained from homologs in other species [52, 53]. PolA was the only protein which links (based on text mining) HU to other beta-lactam resistance proteins such as penicillin-binding protein I (PbpA) (interaction score: 0.499) and beta-lactamase (BlaZ) (interaction score: 0.425) [52,54]. PbpA was also shown to share the common interactor DivIB with discriminant proteins RpmD and RpmJ. ErmA was shown to share common nodes (ribosomal proteins) with the discriminant proteins RpmD and RpmJ. ErmA was shown, based on text mining, to also interact with PolA, linked to HU as previously described, (interaction score: 0.611) [55] and to other proteins (RpsA, MetG and GuaA), based on co-expression, gene fusion and co-occurrence (interaction scores >0.400). NorA was shown to share a common interactor, DNA topoisomerase (TopA) with the discriminant protein HU. ABC-2 was shown to share common interactors, signal recognition particle proteins FfH and FtsY with discriminant proteins RpmD and RpmJ. MBL was shown to share a common interactor, putative fatty oxidation complex protein (AID38649.1), with discriminant protein RpmJ based on co-expression, gene fusion and co-occurrence (interaction scores > 0.400).

Notably, the PPI analysis of the benzylpenicillin-resistant proteome, 153 proteins–a total of 4 discriminant proteins and 149 first neighbour proteins–showed higher connectivity (clustering coefficient 0.728) than the complete *S*. *aureus* proteome network (clustering coefficient 0.421). The average number of neighbours per protein was 68.719 in the benzylpenicillin-resistant proteome network and 27.190 in the complete *S*. *aureus* proteome network. In terms of network density, the values ranged between 0.452 (benzylpenicillin-resistant proteome network) and 0.009 (complete *S*. *aureus* proteome network) and for the network heterogeneity the values ranged between 0.528 benzylpenicillin-resistant proteome network) and 1.243 (complete *S*. *aureus* proteome network).

## Discussion

Antibiotic-resistant *S*. *aureus* infections are a major concern in human and veterinary medicine. Recently, dairy cattle have been shown to be an important risk factor for zoonotic transfer [1]. Fast, affordable and effective diagnostic solutions which are able to detect the specific *S*. *aureus* strains and their antibiotic resistance and susceptibility profiles are key to support effective and targeted treatment selection.

Motivated by identifying the most effective method to discriminate (MDR- and benzylpenicillin-) resistant and susceptible *S*. *aureus* strains, we approached the task in a principled way by applying optimization techniques to overcome uncertainty in data features and by using a wide repertoire of classification methods. In general, most of the classifiers tested achieved high performance and had kappa values over 85.00%. However, amongst the investigated machine learning approaches RBF SVM, neural network and logistic regression were those that achieved the best performance. Diagnostic systems trained on individual isolates coming from 24 different farms achieved up to (mean result ± standard deviation over 30 runs on the test set): accuracy = 97.54% ± 1.91%, sensitivity = 99.93% ± 0.25%, specificity = 95.04% ± 3.83%, and kappa = 95.04% ± 3.83% in RBF SVM algorithm. We showed that our classification methods while offering high out-of-sample accuracy can also be solved in practical computational times.

While our primary aim was to develop machine learning-powered diagnostics discriminating benzylpenicillin-resistant and susceptible isolates of bovine mastitis-causing *S*. *aureus*, we also characterized the molecular determinants and interactions underlying the identified antibiotic resistance and susceptible patterns. Several isolates were obtained from the same animal, some of them also presented the same antimicrobial susceptibility profile, possibly suggesting that they represent the same strain. Moreover, none of the *S*. *aureus* isolates, except one, were found resistant to cefoxitin or oxacillin, despite being resistant to penicillin, suggesting that penicillin-resistant *S*. *aureus* isolates in this study were maybe indeed producers of penicillinase instead of being MRSA. This might be related to the fact that since the first report of *S*. *aureus* resistant to methicillin detected in a dairy herd in the United Kingdom [12] and from the first isolation in 2012, of both *mecA* and *mecC* LA-MRSA in bulk milk from dairy cattle in the UK [17], frequency of detection of *mecA* and *mecC* LA-MRSA in the UK, gathered from surveillance and large-scale dairy cattle studies, [11,17] remained low [15]. The low frequency of resistance to cefoxitin or oxacillin found in our cohort is possibly reflecting that LA-MRSA is present in the UK, possibly at a low prevalence level.

Our findings showed that the five MALDI-TOF peaks recognized as significant by the trained classifiers were found to correspond to two ribosomal proteins (RpmJ and RpmD), DNA-binding HU protein and two hypothetical proteins. RpmD, DNA-binding HU protein and two hypothetical proteins were also found to give the best discrimination between multidrug-resistant and susceptible profiles of *S*. *aureus*.

The notion that components of the ribosome are important in the growth rate and antibiotic resistance of bacteria is a well-known concept [56]. Among those determinants involved in intrinsic resistance, ribosomal proteins have been found to deal with the general response to stress [57]. Similarly, recent findings highlighted the existence of ribosomal mutations conferring resistance to antibiotics of several classes not targeting the ribosome [56]. Specifically, it has been shown that ribosomal mutations can contribute to the evolution of multidrug-resistant profiles, by inducing ribosomal mis-assembly, that in turn leads to a systematic transcriptional cell alteration, ultimately impacting resistance to multiple antibiotics by interfering with different cellular pathways [56]. *RpmJ* was shown to be up-regulated in *Pseudomonas aeruginosa* when treated with ciprofloxacin and fluoroquinolone [58] and similarly in *S*. *epidermidis* [59]. Moreover, *rpmJ* was shown to confer intrinsic multidrug resistance to a varied set of antibiotics (nitrofurantoin, sulfamethoxazole, rifampicin, tetracycline, vancomycin, ampicillin, colistin, erythromycin) in *E*. *coli*, where deletion of this gene caused the bacteria to become more sensitive than wild type [60]. In comparison, fewer literature works have been published about *rpmD* and antibiotic resistance. Sharma-Kuinekel and collaborators showed that *rpmD* was downregulated in *S*. *aureus* strains which had the antibiotic tolerance related LytSR system silenced [61].

The discriminant protein DNA-binding HU protein was found essential in the bacterial survival and growth of *S*. *aureus* [62]. It was also previously found to be correlated to antibiotic resistance by being upregulated in the mutant *S*. *aureus* isolates with silenced serine/threonine kinase PknB, which also has a penicillin-binding domain [63]. Besides the proteins with known functions, we also identified two hypothetical proteins, but we were unable to find any evidence so far linking them to antibiotic resistance. Although it was not possible for us to identify the function of these hypothetical proteins, by applying PSI-BLAST and PSORTb v3.0 together with 3D threading modelling searches, the hypothetical proteins are predicted to be involved in pathways such as antibiotic metabolic process, lipid/protein transport and ion binding.

Although the elected mechanism to acquire resistance in *S*. *aureus* is through horizontal gene transfer, spontaneous mutations in the core genome and positive selection are also mechanisms used by the bacteria to acquire several resistances (e.g., fluoroquinolones, linezolid and daptomycin) [27]. The spontaneous mutation mechanisms involving ribosomal proteins in *S*. *aureus* has been previously found to raise antibiotic resistance (e.g. vancomycin) [64]. Future efforts may integrate genome sequencing analysis of the isolated strains towards elucidating and understanding the mechanisms underlying the antibiotic resistance.

We were not surprised that known genes such as *blaZ*, *mecA*, *pbpA*, conferring resistance to penicillin in *S*. *aureus* were not amongst the MALDI-TOF peaks recognized as significant by the trained classifiers. This is because the mass range resolution of the MALDI-TOF was set to be between 2kDa and 12kDa, and the BlaZ, MecA, PbpA are all proteins with molecular weights higher than 20kDa. However, our PPI cluster analysis results showed that these proteins known to confer resistance have all been found to interact with most of the proteins corresponding to the MALDI-TOF peaks and to form a highly connected benzylpenicillin proteome network.

While our approach successfully developed a diagnostic solution to identify antibiotic-resistant signatures, there are limitations to our method which future work may build upon. For one, the working range of 2-12kDa does not give the possibility to study the complete *S*. *aureus* proteome in relation to a specific phenotype.

The MDR and XDR isolates, collectively named multidrug-resistant isolates, used in this study were all resistant to benzylpenicillin in addition to other antimicrobial agents. Therefore, there is a bias towards peaks determining resistance or susceptibility to benzylpenicillin, which may explain why all 4 multidrug discriminant peaks occurred within the set of benzylpenicillin-only discriminant peaks.

In this work, we have opted to pre-process all the data together as previously done by several studies [42,65–68] instead of splitting it into a training and validation sets for several reasons. First, given the low number of samples in each of the two minority classes (multidrug resistant and benzylpenicillin-only resistant) it would have been not possible to have a sufficient number of observations in each set and each partition being enough representative to yield a good peak selection. Moreover, because some of the peaks appeared in just a subset of these samples (minority classes), the random sampling of the data performed could increase the chances of getting spurious peaks in the training set that would not represent the whole minority class. To avoid these problems, we pre-processed all the data together.

Moreover, this study has been confined to a relatively small number of isolates. Ideally, a larger number of isolates would have allowed to refine the machine learning predictions. However, other studies attempted the analysis of antimicrobial resistance on *S*. *aureus* with MALDI-TOF and machine learning and similar sample size. For example, Tang *et al*. [39], to implement heterogenous VISA (hVISA) detection models, examined 10 MSSA and 10 MRSA clinical isolates recovered from individual patients. Wang *et al*. [40], used MALDI-TOF mass spectra obtained from 35 hVISA/ VISA and 90 VSSA isolates. Mather *et al*. [37], tested 21 VISA, 21 hVISA, and 38 VSSA isolates to develop their SVM based models. Usually, the larger the dataset the greater is the statistical power for pattern recognition. However, in our machine learning approach, we have used the Nested CV approach which is known to produce robust and unbiased performance estimates regardless of sample size [69]. The machine learning performance indicators associated with our models are high suggesting that models were sufficiently trained.

In addition, we acknowledge, as a limitation of this study, that our data were collected from farms only in England and Wales. However, this should not pose a restriction on our method’s ability to predict resistance or susceptibility in other farms across the globe. If it is given a sufficiently diverse distribution of data to train the supervised learning algorithms, this would reduce any geographical bias that could affect predictive capability. This study should be considered a proof-of-principle where we conducted a feasibility work to invest on with larger samples and geographical areas.

Finally, the downside of requiring larger sample sizes is limitations in data availability, often requiring reliance on public databases and thus compromise on the type of available data and possible studies. Unfortunately, in omics and other technology-based data collection analysis, very often only small samples are available, this is because of limited in vivo experiments, protocols, involvement of human participants and costs. For example, whilst not being able to rely on large amounts of data, we had the unprecedented possibility to demonstrate that our methodology is associated with high classification accuracy even when using small sample size, this applicability may facilitate research scenarios where only limited data is available.

In addition to the machine learning analyses undertaken to prove the effectiveness of our method to differentiate susceptibility/resistance profiles rather than strain differences, we also compared the MALDI-TOF spectral peaks spectral peaks (4305.59Da, 4807.21Da, 6422.3Da, 6891.17Da and 9621.26Da) recognised as discriminant by our trained classifiers with the peaks previously found in literature to discriminate the main clonal lineages of *S*. *aureus* [41,44–47]. When we compared our peaks with those found by Wolters *et al*. [45], Böhme *et al*. [46] and Camoez *et al*. [47], no common peaks were found between the studies. However, similarities were found between our results and the findings reported by Josten *et al*. [44] and Lasch *et al*. [70].

In particular, the peaks at *m/z* 4305.59 (RpmJ), 6422.37 (RpmD), 6891.17 (HP2) and 9621.26 (DNA binding protein HU) were revealed to be in common between our study and Josten *et al*. [44]. However, the variant (*m/z* 6397) of the ribosomal protein RpmD found by Josten *et al*. [44] to be discriminant for the subgroup of CC22 strains was not present in our spectra as we only detected the peak at *m/z* 6422.37 corresponding to RpmD. Moreover, although the protein RpmD was considered a biomarker by Josten *et al*. [44], it only showed a limited sensitivity (0.167), reflecting a low level of conservation of the mutations in the clonal lineages. For example, the CC22 biomarker was not conserved in all spa types of this clonal complex [44]. The peaks *m/z* 4305.59 (4306 in Josten *et al*. [44]), 6891 (6889 in Josten *et al*. [44]) and 9621.26 (9627 in Josten *et al*. [44]) although identified in the *S*. *aureus* spectra by Josten *et al*. [44] were not included in the list of markers distinguishing the different strains. Moreover, Lasch *et al*. [70] analysed 59 diverse *S*. *aureus* isolates from 6 different lineages using MALDI-TOF mass spectrometry. Based on their results over a gel view representation and a hierarchical cluster analysis, the authors indicated that, with a few exceptions, CC-specific biomarkers for *S*. *aureus* are an exception rather than a rule. The authors found 3 regions that could be considered biomarkers for some lineages: *m/z* 3875 and 3891 (CC5); *m/z* 6552 and 6592 (CC8); *m/z* 5002 and 5032 (CC22). Therefore, none of the peaks used in our study were considered biomarkers by Lasch *et al*. [70]. The results found by Lasch *et al*. [70] clearly suggests that typing *S*. *aureus* can be rather unsuccessful due to a lack of stable biomarkers to distinct clonal groups, a low classification accuracy based on different CC types and a cluster analysis that indicate the limited possibilities to differentiate *S*. *aureus* below species levels.

Further comparisons were also made with existing literature coupling MALDI-TOF mass spectrometry with a refined analysis framework to accurate classify resistant and susceptible *S*. *aureus* strains. In particular, the peaks (*m/z* 4305.59, 4807.21, 6422.3, 6891.17 and 9621.26) recognised as discriminant for the susceptible and resistant profiles in this study with those previously found [36, 39] differentiating MSSA and MRSA recovered from clinical samples or at distinguishing VSSA from hVISA/VISA [37,40] no similar peaks were detected under the experimental conditions chosen here. In particular, our peaks often mapped in the higher and non-overlapping mass range of the spectrum. Whereas, when we compared our peaks with those found by Asakura *et al*. [41] to differentiate VISA, hVISA, and VSSA clinical isolates, we found that one peak (*m/z* 4306) was in common between the two studies. This peak is among 23 other peaks that were found to be statistically significant among VISA, hVISA and VSSA (p < 10^−4^, Kruskal-Wallis test). This peak corresponds to the ribosomal protein RpmJ. Indicating that ribosomal proteins can be correlated with resistance phenotypes. This was also reported by Josten *et al*. [44] when analysing the peak pattern of 401 MRSA and MSSA strains (see above).

Although we have not typed our strains, which we acknowledge as a limitation of our study, we believe that it is not unreasonable to assume that we have classified the resistance/susceptibility phenotype and not the strains. Our supervised learning-based classifier consisted of a binary classification (resistant/susceptible), where each observation (isolate) was labelled according to the MIC values obtained for each specific isolate. Given the high performance indicators accompanying our classification and given the variety of different peaks among strains as shown by Josten *et al*. [44], Wolters *et al*. [45], Böhme *et al*. [46], Camoez *et al*. [47] and Lasch *et al*. [70], it is very unlikely that we could separate all the different strains circulating in just two groups and importantly with such high performance indicators. From a machine learning point of view, given the limited number of observations, relative high number of possible strains, binary outcome, number of genetic/molecular traits different among the strains it would not had been possible to separate the different strains in just two groups especially with such high-performance scores. This is also in agreement with Lasch *et al*. [70] that although performing an elegant modular/hierarchical ANN analysis of spectra from the *S*. *aureus* data set (we only did a one-step machine learning classification), apart from a fairly good classification accuracy for CC8 strains of *S*. *aureus* and, to a lesser extent for strains of CC5 (80%) and CC30 (78%), the classification accuracy for the other strains was unacceptably low. Despite intensive efforts aiming at improving these outcomes, neither variations of the spectral pre-processing nor of the network topology resulted in better classification results according to the authors.

Overall, we demonstrated that the combination of supervised machine learning and MALDI-TOF mass spectrometry can be used to develop an effective computational diagnostic solution that can discriminate between benzylpenicillin/multidrug-resistant and susceptible *S*. *aureus* strains. Our solution could save time and money with respect to traditional susceptibility testing which is not viable for day-to-day monitoring of antibiotic resistance. Our solution could support farmers with timely, accurate and targeted treatment selection.

## Methods

### Ethics statement

This study received an ethical review and approval from the Clinical Ethical Review panel at the School of Veterinary Medicine and Science, University of Nottingham (approval Reference number: 2067 170717). All data is owned by QMMS ltd.

### Data source

82 *S*. *aureus* isolates were collected from 67 animals that were diagnosed with bovine mastitis in 24 different farms, in England and Wales between March 2004 and May 2005. The animals with mastitis were either primiparous (n = 9) or multiparous (n = 73, median parity = 4). On the day of sample collection, the days in milk of the cows varied from 1 to 569 days with a median value of 160 days.

### Sample analysis

Bovine mastitis-causing *S*. *aureus* isolates were tested on VITEK 2 AST-GP79 using one Antibiotic Susceptibility Testing (AST) card per isolate. Each card was filled with at least one positive control well with no antibiotic and multiple wells with increasing concentrations of antibiotics. We tested susceptibility to the following antibiotics: benzylpenicillin, cefoxitin, oxacillin, cefalotin, ceftiofur, cefquinome, amikacin, gentamicin, kanamycin, neomycin, enrofloxacin, clindamycin, erythromycin, tilmicosin, tylosin, tetracycline, florfenicol and trimethoprim/sulfamethoxazole. Using the VITEK 2 we measured the growth and viability of the isolates in all wells compared to the control wells. Relative bacterial growth in each antibiotic well was calculated and compared with the positive control wells. The minimum inhibitory concentration (MIC) values were calculated by comparing the growth of the bacteria to the growth of isolates with known MICs. The *S*. *aureus* isolates were labelled as either resistant or susceptible according to their antibiotic resistance profiles based on CLSI breakpoints (VET01-S3) [71].

### Generation of MALDI-TOF spectra

All *S*. *aureus* isolates were stored at -80°C since their recovery in 2004/5 using a microbead preservation system (Technical Service Consultants Ltd, Lancashire). Isolates were recovered onto Blood agar and incubated at 37°C for 24 hours. If no growth was initially observed the isolates were sub-cultured another 24 hours. All isolated were sub-cultured on blood agar at 37°C for 24 hours prior to MALDI-TOF analysis. The same storage and growth conditions were applied to all isolates.

The pure cultures were then analysed using the Time-of-flight (TOF) MALDI mass spectrometer (Bruker Daltonics, Billerica, MA), Microflex–Flex Control Version 3.4, Bruker Daltonics. The order of sample analysis was randomised, the Bruker Bacterial Test Standard (BTS) (Bruker Daltonics) was used for calibration control on every plate. For each isolate, six technical replicates were generated from 240 desorption’s per replicate (6 x 40 shots), and protein mass spectra acquired in the range 2000 to 20,000 Da were generated. Spectra were compared visually using Biotyper 3.1 (Bruker Daltonics) to remove low intensity spectra or spectra with substantial background noise. All the samples used in this study were further analysed visually on Matlab for insufficient resolution (defined as a measure to distinguish two peaks of slightly different *m/z* values [72]), low intensity or substantial background. However, no samples were discarded for these reasons. The. Technical replicates were further compared using composite correlation indices (CCI) to remove dissimilar spectra with CCI < 0.99 [73]. At least three good quality spectra per isolate were required for inclusion of the isolate in the analysis. Moreover, when three qualifying technical replicates could not be obtained the sample was re-analysed in order to get at least 3 replicates. All the 82 isolates used in this study had three good quality technical replicates.

### Data processing

The pre-processing steps of MALDI-TOF mass spectra were performed using MATLAB Bioinformatics Toolbox Release 2017b, The MathWorks, Inc., Natick, Massachusetts, United States. Our analysis was done using 82 *S*. *aureus* isolates with each sample having 3 to 6 replicates.

The pre-processing followed these 8 steps:

1. **Mean Computing:** the replicates of each biological isolate were averaged.
2. **M/Z Cropping:** the mass range was cropped to be between 2kDa and 12kDa.
3. **Resampling:** the data was up-sampled from 13,740 to 20,000 points.
4. **Baseline Correction:** for each biological isolate, baseline correction was applied by using a window of 200 Da with a step size of 200 Da to shift the window. The quantile method (10% value) was used to find the likely baseline value in every window. Shape-preserving piecewise cubic interpolation approximation was applied to regress the varying baseline. The regressed baseline was not smoothed. The resulting baseline was subtracted from the spectrum.
5. **Normalisation:** the area under the curve (AUC) of every spectrum was normalised to the median and post-rescaled such that the maximum intensity was 100.
6. **Noise reduction:** each sample was denoised using least-squares polynomial with a window of 35 Da and a 2-degree polynomial function.
7. **Alignment:** to align the spectrograms, a set of reference peaks was required. Specifically, the peaks were selected if present in at least 30% of all spectra. The 30% threshold was chosen following the workflow suggested in the ClinProTools software documentation [74]. In addition, the first pre-processing step of our workflow consists of averaging all the 3 or more technical replicates of each sample. Therefore, after this averaging step we have one spectrum per sample and consequently the 30% threshold used to select the peaks is applied to all samples. By applying the 30% threshold we are selecting only the peaks that are present and hence relevant across both the resistant and susceptible classes, as shown in Tables 1 and 2 in the Results section. The alignment was estimated using the default values of msalign function (Bioinformatics Toolbox).
8. **Peak Detection:** To retain a reasonable intensity a signal-to-noise ratio threshold was defined at 10% to discard all peaks below it. Therefore, since the spectra were previously normalised to an overall maximum intensity of 100, any point below 10 is considered noise. A minimum distance of 20Da between neighbouring peaks was set, i.e., two peaks must be at least 20Da apart to be considered different.

### Spectral features

After detecting all the peaks in each spectrum, a peak list report was prepared similarly to ClinProTools 3.0 [74]. Specifically, the peaks were selected if present in at least 30% of all spectra. The selected peaks were further pre-processed to have zero mean and unit variance. Such peaks represented the spectral features used in the classification analysis.

### Classification methods

The performance of the classifiers, naïve Bayes [75], linear and non-linear (RBF kernel) support vector machines (SVM) [76], decision tree [77], random forests [78], multi-layer perceptron neural networks (MLP) [79], AdaBoost (AdaBoost-SAMME version [80]), logistic regression [81], linear discriminant analysis (LDA) [82] and quadratic discriminant analysis (QDA) [82], was investigated using the scikit-learn library in Python [83].

For the classifiers, the following set of values were employed for the hyper-parameter searches:

- Logistic Regression: inverse of regularization strength C = [0.001, 0.01, 0.1, 1, 10, 100, 1000].
- Linear SVM: penalty parameter of the hinge loss error C = [0.001, 0.01, 0.1, 1, 10, 100, 1000].
- Decision tree: maximum depth of tree = [10, 20, 30, 50, 100].
- Random Forests and Adaboost: Number of estimators = [2, 4, 8, 16, 32, 64].
- MLP Neural Network: α (L2 penalty parameter) = [0.001, 0.01, 0.1, 1, 10, 100], learning rate (initial learning rate used to control the step size in updating the weights with adam solver) = [0.001, 0.01, 0.1, 1] and hidden layer sizes = [10, 20, 40, 100, 200, 300, 400, 500].
- Non-linear SVM with RBF kernel: γ (RBF kernel coefficient) = [0.0001, 0.001, 0.01, 0.1] and C (L2 penalty parameter) = [0.001, 0.01, 0.1, 1, 10, 100, 1000].
- Naive Bayes, LDA and QDA do not have hyper-parameters.

### Prediction performance

The prediction performance of each classifier was evaluated by considering the following indicators, assuming P and N as the total number of positive (benzylpenicillin/multidrug-resistant) and negative (multidrug susceptible) isolates, respectively and using T for true (correct) and F for false (wrong) predictions:

- Sensitivity (True Positive Rate) = TP / P
- Specificity (True Negative Rate) = TN / N
- Accuracy = (TP+TN)/(P+N)
- Kappa = (*p*_*o*_*−p*_*e*_)/(1-*p*_*e*_) where *p*_*o*_ = (TP+TN)/(P+N) and p_*e*_ = (P*(TP+FN) + N*(FP+TN)) /(P+N)^2^

### Performance analysis

Nested Cross-validation (NCV) [84], which is a well-established cross-validation technique was employed to assess the performance and select the hyper-parameters of the proposed classifiers.

In NCV there is an outer loop split of the data set into test and training sets. For each training set, a grid search (inner loop) is run, in order to find the best hyper-parameters of the classifier using accuracy as a performance metric. Then, the test set is used to score the best classifier found in the inner loop. These scores tell us how well the classifier model generalises, given the best hyper-parameters found in the inner loop.

Thirty iterations were carried out, wherein each iteration an NCV was employed. The inner loop of the NCV finds the best hyper-parameters of each classifier (when suited) using a stratified 3-fold cross-validation; the outer loop measures the accuracy, sensitivity, specificity and kappa using a 5-fold stratified cross-validation, in order to compare all the classifiers [85].

### Biomarker characterization–identification of the protein corresponding to MALDI-TOF spectral peaks recognised as discriminant by the trained classifiers

A dedicated bioinformatics pipeline was developed to find correspondences between individual peaks selected by the machine learning-based classifiers and actual proteins of *S*. *aureus*. First, amino acid sequences of the proteins in the *S*. *aureus* Newbould 305 (ATCC 29740) proteome, which is considered the model bovine mastitis strain [86], were retrieved from the PATRIC database in FASTA format. The molecular weights of the proteins were calculated using the Compute pI/Mw tool on ExPASy [87]. The proteins were filtered in the range of ± 200Da of the mass of individual peaks. Then, N-terminal methionine cleavage was predicted using the online prediction tool TermiNator [88] and the theoretical molecular weights of the proteins were re-calculated using compute pI/Mw tool according to presence or absence of the initial methionine. Finally, proteins with a maximum of 0.2% difference in mass to the individual peaks for the successful identification of correspondence were selected.

To further investigate the function of the identified proteins, we studied protein-protein interactions (PPI) as previously described [89]. The PPI dataset of *S*. *aureus* (strain NCTC 8325/PS 47) was obtained from the STRING database [90] and nodes (proteins) with interaction scores lower than medium confidence level (interaction scores <0.400) were filtered out. The remaining nodes (proteins) were analysed in Cytoscape 3.7.1 based on the following parameters: the average number of neighbours, clustering coefficient, network density and network heterogeneity [91–93].

The characterisation of antibiotic-resistant genes of the beta-lactam, macrolide and tetracycline antibiotic classes in the PPIs, were obtained from ResFinder v3.1 [94] and using them as queries in a comparative BLAST search against the *S*. *aureus* proteome. The functions of the genes in the network were annotated with Gene Ontology terms (biological process, molecular function and cellular component) and KEGG pathways. Finally, to gain a more in-depth understanding of the protein functions, homology and threading 3D models for discriminant proteins were built. 3D homology modelling was used for the proteins with good quality templates in the Swiss-Model repository [95] and the models built by using Swiss-PdbViewer [96]. The 3D models of hypothetical proteins were generated by using the threading technique on I-TASSER, where biological functions were predicted as well [97]. The 3D Models of all discriminant proteins were visualized and edited in UCSF Chimera [98].

Homologs of the discriminant proteins were checked in the NCBI database by position-specific iterative basic local alignment tool (PSI-BLAST). Functional domains were searched against the CDD v3.17–52910 PSSMs database. PSORTb v3.0 was used to predict cellular locations of the discriminant proteins [99].

## Supporting information

S1 Table Breakdown of samples per farm. (XLSX)

S2 Table Antimicrobial susceptibility profile of the resistant isolates that were obtained from the same animal. (XLSX)

S3 Table A) Supervised machine learning prediction of multidrug resistance spectral signature profiles using the Linear Discriminant Analysis (LDA) classifier. Prediction performance results using all the peaks (4807m/z, 6422m/z, 6891m/z and 9621m/z); only the non-ribosomal peaks (4807m/z, 6422m/z, 6891m/z and 9621m/z) and only the ribosomal peak (6422m/z). B) Supervised machine learning prediction of multidrug resistance spectral signature profiles using a non-linear (RBF kernel) support vector machine (RBF-SVM) classifier. Prediction performance results using all the peaks (4305m/z, 4807m/z, 6422m/z, 6891m/z and 9621m/z); only the non-ribosomal peaks (4807m/z, 6422m/z, 6891m/z and 9621m/z) and only the ribosomal peak (4305m/z and 6422m/z).
(DOCX)
