# Supplementary material for: Mass spectrometry and machine learning for the accurate diagnosis of benzylpenicillin and multidrug resistance of Staphylococcus aureus in bovine mastitis
Source: PLoS Comput Biol. 2021 Jun 11;17(6):e1009108. doi: 10.1371/journal.pcbi.1009108 (PMC8221797; doi:10.1371/journal.pcbi.1009108)
Supplement: S3 Table — A) Supervised machine learning prediction of multidrug resistance spectral signature profiles using the Linear Discriminant Analysis (LDA) classifier. Prediction performance results using all the peaks (4807m/z, 6422m/z, 6891m/z and 9621m/z); only the non-ribosomal peaks (4807m/z, 6422m/z, 6891m/z and 9621m/z) and only the ribosomal peak (6422m/z). B) Supervised machine learning prediction of multidrug resistance spectral signature profiles using a non-linear (RBF kernel) support vector machine (RBF-SVM) classifier. Prediction performance results using all the peaks (4305m/z, 4807m/z, 6422m/z, 6891m/z and 9621m/z); only the non-ribosomal peaks (4807m/z, 6422m/z, 6891m/z and 9621m/z) and only the ribosomal peak (4305m/z and 6422m/z). (DOCX) [file pcbi.1009108.s003.docx]

**Mass spectrometry and machine learning for the accurate diagnosis of benzylpenicillin and multidrug resistance of *Staphylococcus aureus* in bovine mastitis**

**Necati Esener^1ξ^, Alexandre Maciel Guerra^2 ξ,^ Katharina Giebel^3^, Daniel Lea^4^, Martin J. Green^1^, Andrew J. Bradley^1,3^and Tania Dottorini ^1*^**

^1^University of Nottingham, School of Veterinary Medicine and Science, College Road, Sutton Bonington, Leicestershire, LE12 5RD, UK

^2^University of Nottingham School of Computer Science, Jubilee Campus, Wollaton Rd, Nottingham, Nottinghamshire NG8 1BB, UK

^3^Quality Milk Management Services ltd, Cedar Barn, Easton Hill, Easton, Wells, BA5 1DU, UK

^4^Digital Research Service, University of Nottingham, College Road, Sutton Bonington, Leicestershire, LE12 5RD, UK

*corresponding author (email: tania.dottorini@nottingham.ac.uk)

**ξ** co-authors

Supplementary Table 3a. Supervised machine learning prediction of multidrug resistance spectral signature profiles using the Linear Discriminant Analysis (LDA) classifier. Prediction performance results using all the peaks (4807m/z, 6422m/z, 6891m/z and 9621m/z); only the non-ribosomal peaks (4807m/z, 6422m/z, 6891m/z and 9621m/z) and only the ribosomal peak (6422m/z).

|  | **All Proteins** | **Non-ribosomal** | **Ribosomal** |
| --- | --- | --- | --- |
| **Accuracy** | 96.81 ± 0.43 | 93.62 ± 1.55 | 77.25 ± 0.00 |
| **Sensitivity** | 99.88 ± 0.41 | 96.07 ± 0.10 | 100.00 ± 0.00 |
| **Specificity** | 95.96 ± 0.52 | 85.33 ± 6.75 | 0.00 ± 0.00 |
| **Cohen's kappa** | 91.83 ± 1.37 | 81.00 ± 5.85 | 0.00 ± 0.00 |

Supplementary Table 3b. Supervised machine learning prediction of multidrug resistance spectral signature profiles using a non-linear (RBF kernel) support vector machine (RBF-SVM) classifier. Prediction performance results using all the peaks (4305m/z, 4807m/z, 6422m/z, 6891m/z and 9621m/z); only the non-ribosomal peaks (4807m/z, 6422m/z, 6891m/z and 9621m/z) and only the ribosomal peak (4305m/z and 6422m/z).

|  | **All Proteins** | **Non-ribosomal** | **Ribosomal** |
| --- | --- | --- | --- |
| **Accuracy** | 97.54 ± 1.91 | 93.14 ± 1.22 | 75.99 ± 1.86 |
| **Sensitivity** | 99.93 ± 0.25 | 95.24 ± 1.18 | 98.24 ± 2.37 |
| **Specificity** | 95.04 ± 3.83 | 86.00 ± 3.59 | 0.44 ± 1.66 |
| **Cohen's kappa** | 95.04 ± 3.83 | 80.38 ± 3.54 | -1.54 ± 3.22 |
