# Supplementary material for: Mass spectrometry and machine learning for the accurate diagnosis of benzylpenicillin and multidrug resistance of Staphylococcus aureus in bovine mastitis
Source: PLoS Comput Biol. 2021 Jun 11;17(6):e1009108. doi: 10.1371/journal.pcbi.1009108 (PMC8221797; doi:10.1371/journal.pcbi.1009108)
Supplement: Attachment — Submitted filename: Response to Reviewers.docx [file pcbi.1009108.s004.docx]

**Response to Reviewers**

We thank the Editor and Reviewers for their useful comments, which no doubt contributed to make this a better paper. In the following, a point-by-point response to all the questions and comments is provided. The original questions are in blue, our replies in black.

Editor’s comments

Although the reviewers both indicate that major revisions are required I do think that this is an interesting study and worthy of publication. I would encourage the authors to address their comments and edit the manuscript accordingly. There may be some comments that you disagree with and so please address these in your response to reviewers. The edited manuscript will be sent out to the same reviewers.

We were delighted that the Editor recognised the research as interesting and worthy of publication.

Items included in this submission:

i. Response to reviewers;
ii. Revised manuscript marked-up copy (Revised Article with Changes Highlighted.docx);
iii. Revised manuscript clean copy (Manuscript.docx);
iv. Supplementary Tables 1, 2 and 3 – new supplementary files with information requested by the reviewers;
v. Figure 1 – updated figure as requested by the reviewer 1;
vi. Figure 2 – new figure replacing the former table 1 on the original submission;
vii. Figure 3 – previously Figure 2 on the original submission (renamed only);
viii. Figure 4 – previously Figure 3 on the original submission (renamed only);
ix. Figure 5 – previously Figure 4 on the original submission, revised the protein names as requested by reviewer 2;
x. Figure 6 – updated figure due to an error found (please see Additional Edits to the Manuscript on page 30 of this file);
xi. Figure 7 – previously Figure 6 on the original submission (renamed only);
xii. Data availability statement.

In the response to the reviewers when mentioning figures, we have referred to the new numbering.

**Reviewer #1**

**General comments**

The manuscript by Necati Esener and colleagues reports on the use of MALDI-TOF to detect antibiotic resistance profiles for benzylpenicillin and MDR *S. aureus* without prior exposure to antibiotics. Although this use of MALDI-TOF has been reported before, this is the first study to my understanding that has used *S. aureus* isolates from bovine mastitis cases. The manuscript is interesting and has the potential to rapidly speed up the appropriate treatment for bovine mastitis. However, I have several major concerns regarding the manuscript that I believe should be addressed before it is suitable for publication. I have divided these concerns by reference to manuscript sections.

We were delighted that the reviewer recognised the research as interesting and potentially useful for treatment of bovine mastitis.

**Introduction:**

• This type of study has been completed previously on *S. aureus* isolates, albeit not one from bovine mastitis cases. This should be addressed in the introduction in more detail.

Thanks for the suggestion. We have updated the introduction to address the reviewer’s comment (see Introduction lines 138-150 and below)

Introduction – lines 138-150: ‘and, in combination with machine learning techniques, larger datasets, a wide range of microbial species identification and complex antimicrobial resistance profile could be analysed faster and more easily and economically, revolutionizing the field of microbiology (Weis *et al*., 2020). *S. aureus* was one of the most frequently studied genera for antimicrobial resistance prediction (Sogawa *et al*., 2017; Mather *et al*., 2016; Bai *et al*., 2017; Asakura *et al*., 2018; Tang *et al*., 2019; Wang *et al*., 2018). Rapid and accurate classification of MRSA and methicillin-sensitive *S. aureus* (MSSA) based on MALDI-TOF spectral of clinical samples was obtained by several studies (Sogawa *et al*., 2017; Bai *et al*., 2017; Tang *et al*., 2019). Analogously, high accuracy results have been obtained when applying machine learning approaches to MALDI-TOF spectral data for the prediction of the broad-spectrum antibiotic vancomycin. In particular, successful separation of vancomycin-intermediate (VISA) from vancomycin-susceptible *S. aureus* (VSSA) on the basis of MALDI-TOF data collected from clinical samples (Mather *et al*., 2016; Asakura *et al*., 2018; Wang *et al*., 2018). Recently, van Oosten and Klein (2020), developed classification models for *S. aureus* which assign the mechanisms of action of antibacterial drugs.

Asakura K, Azechi T, Sasano H, Matsui H, Hanaki H, *et al.* (2018) Rapid and easy detection of low-level resistance to vancomycin in methicillin-resistant Staphylococcus aureus by matrix-assisted laser desorption ionization time-of-flight mass spectrometry. PLOS ONE 13(3): e0194212. <https://doi.org/10.1371/journal.pone.0194212>

Bai, J., Z. Fan, L. Zhang, X. Y. Xu and Z. Zhang. “Classification of Methicillin-Resistant and Methicillin-Susceptible Staphylococcus Aureus Using an Improved Genetic Algorithm for Feature Selection Based on Mass Spectra.” *ICBBT '17* (2017).

Mather CA, Werth BJ, Sivagnanam S, SenGupta DJ, Butler-Wu SM. Rapid Detection of Vancomycin-Intermediate Staphylococcus aureus by Matrix-Assisted Laser Desorption Ionization-Time of Flight Mass Spectrometry. J Clin Microbiol. 2016 Apr;54(4):883-90. doi: 10.1128/JCM.02428-15. Epub 2016 Jan 13.

Sogawa K, Watanabe M, Ishige T, Segawa S, Miyabe A, Murata S, Saito T, Sanda A, Furuhata K, Nomura F. Rapid Discrimination between Methicillin-Sensitive and Methicillin-Resistant Staphylococcus aureus Using MALDI-TOF Mass Spectrometry. Biocontrol Sci. 2017;22(3):163-169. doi: 10.4265/bio.22.163.

Tang W, Ranganathan N, Shahrezaei V, Larrouy-Maumus G (2019) MALDI-TOF mass spectrometry on intact bacteria combined with a refined analysis framework allows accurate classification of MSSA and MRSA. PLOS ONE 14(6): e0218951. <https://doi.org/10.1371/journal.pone.0218951>

Van Oosten, LN and Klein, CD. Machine Learning in Mass Spectrometry: A MALDI-TOF MS Approach to Phenotypic Antibacterial Screening J. Med. Chem. 2020, 63, 16, 8849–8856 <https://doi.org/10.1021/acs.jmedchem.0c00040>

Wang HY, Chen CH, Lee TY, Horng JT, Liu TP, Tseng YJ, Lu JJ. Rapid Detection of Heterogeneous Vancomycin-Intermediate Staphylococcus aureus Based on Matrix-Assisted Laser Desorption Ionization Time-of-Flight: Using a Machine Learning Approach and Unbiased Validation. Front Microbiol. 2018 Oct 11;9:2393. doi: 10.3389/fmicb.2018.02393.

Weis CV, Jutzeler CR, Borgwardt K. Machine learning for microbial identification and antimicrobial susceptibility testing on MALDI-TOF mass spectra: a systematic review. Clin Microbiol Infect. 2020 Oct;26(10):1310-1317. doi: 10.1016/j.cmi.2020.03.014. Epub 2020 Mar 23.

**Methodology:**

• There is little information on isolation of *S. aureus* isolates. What media was used, growth conditions etc? Were they stored in ultra-low temperature freezers and for how long? Did this differ between collection groups or resistance groups?

As correctly pointed out by the reviewer this information was indeed missing in the manuscript. We have now edited the Methods sections (See Methods – lines 620-624 and below).

Methods – lines 616-620: “All *S. aureu*s isolates were stored at -80 °C since their recovery in 2004/5 using a microbead preservation system (Technical Service Consultants Ltd, Lancashire). Isolates were recovered onto Blood agar and incubated at 37 °C for 24 hours. If no growth was initially observed the isolates were sub-cultured another 24 hours. All isolated were sub-cultured on blood agar at 37 °C for 24 hours prior to MALDI-TOF analysis. The same storage and growth conditions were applied to all isolates.”

• There is too little information on MALDI-TOF acquisition. What instrument was used? I imagine that it was a BioTyper but generic reference to MALDI-TOF is misleading. Were samples randomised during running, including randomisation of technical repeats? What was used as a calibrant control? Although there is reference to a previous publication, in my opinion this information should be fully reported in the manuscript.

Again, thanks for the useful suggestion, as this part was indeed missing. We have now updated the Methods (See Methods lines 621-635 and below)

Methods – lines 621-635: The pure cultures were then analysed using the Time-of-flight (TOF) MALDI mass spectrometer (Bruker Daltonics, Billerica, MA), Microflex – Flex Control Version 3.4, Bruker Daltonics. The order of sample and technical replicate analysis was randomised, the Bruker Bacterial Test Standard (BTS) (Bruker Daltonics) was used for calibration control on every plate. For each isolate, six technical replicates were generated from 240 desorption’s per replicate (6 x 40 shots), and protein mass spectra acquired in the range 2000 to 20,000 Da were generated. Spectra were compared visually using Biotyper 3.1 (Bruker Daltonics) to remove low intensity spectra or spectra with substantial background noise. All the samples used in this study were further analysed visually on Matlab for insufficient resolution (defined as a measure to distinguish two peaks of slightly different *m/z* values (Mulvaney, 1993)), low intensity or substantial background. However, no samples were discarded for these reasons. Technical replicates were further compared using composite correlation indices (CCI) to remove dissimilar spectra with CCI < 0.99 (Archer *et al*., 2017). At least three good quality spectra per isolate were required for inclusion of the isolate in the analysis. Moreover, when three qualifying technical replicates could not be obtained the sample was re-analysed in order to get at least 3 replicates. All the 82 isolates used in this study had three good quality technical replicates.

Archer SC, Bradley AJ, Cooper S, Davies PL, Green MJ. Prediction of Streptococcus uberis clinical mastitis risk using Matrix-assisted laser desorption ionization time of flight mass spectrometry (MALDI-TOF MS) in dairy herds. Prev Vet Med. 2017 Sep 1;144:1-6. doi: 10.1016/j.prevetmed.2017.05.015. Epub 2017 May 17. PMID: 28716189; PMCID: PMC5529212.

R.L. Mulvaney (1993), Mass Spectrometry, In R. Knowles (Ed.), T. H. Blackburn (Ed.), Nitrogen Isotope Techniques, Academic Press, pp. 11-57, ISBN 9780124169654, https://doi.org/10.1016/B978-0-08-092407-6.50007-9.

• The authors states that technical replicates with an insufficient resolution were removed. What were the criteria for this? Was this random or related to sample source etc?

Please see also the answer to the previous question. The major criteria to discard samples was composite correlation indices (CCI) to remove dissimilar spectra with CCI < 0.99 (Archer *et al*., 2017). However, on Matlab we have double-checked the resolution of all the samples on this study. Specifically, the resolution is defined as a measure to distinguish two peaks of slightly different *m/z* values (Mulvaney, 1993). No samples were discarded due to this metric.

We have now added this information in the methods section (See Methods – lines 621-635)

R.L. Mulvaney (1993), Mass Spectrometry, In R. Knowles (Ed.), T. H. Blackburn (Ed.), Nitrogen Isotope Techniques, Academic Press, pp. 11-57, ISBN 9780124169654, https://doi.org/10.1016/B978-0-08-092407-6.50007-9.

• 82 isolates were started with and 82 were used in data processing. The authors refer in line 419-420 that isolates with less than three technical replicates were removed. Did this happen?

The Reviewer is correct, at least three good quality replicates were required for inclusion of the isolate in the analysis. However, all the isolates used in this study had more than 3 good quality replicates, so no isolates were removed because of this selection criterion. Moreover, when three qualifying technical replicates could not be obtained the sample was re-analysed in order to get at least 3 replicates. Therefore, as correctly pointed out by the reviewer in this study all isolates were started and used. We have updated the Method section line 632-635 to better clarify this concept.

Method - Line 632-635: “At least three good quality spectra per isolate were required for inclusion of the isolate in the analysis. Moreover, when three qualifying technical replicates could not be obtained the sample was re-analysed in order to get at least 3 replicates. All the 82 isolates used in this study had three good quality technical replicates”

• Spectral peak selection: authors refer in line 450 that peaks selected if present in at least 30% of all spectra. Is this 30% of samples or 30% of all generated spectra? Why was a cut-off chosen – could this remove peaks that differentiated between susceptible/resistant isolates as the group sizes were not balanced?

We have applied a 30% spectral peak selection procedure for two reasons. First, the 30% threshold was chosen following the workflow suggested in the ClinProTools software documentation (ClinProTools 3.0: User Manual, 2011). Second, the first pre-processing step of our workflow consists of averaging all the 3 or more technical replicates of each sample. Consequently, after this averaging step we have one spectrum per sample and therefore the 30% threshold to select the peaks is applied to all samples. We agree with the Reviewer that this cut-off could possibly remove some peaks that differentiated between susceptible and resistant; especially if a specific peak appeared in the minority class (resistant) and not in the majority class (susceptible). However, in this case scenario, we expect these peaks not to be representative of the whole minority (resistant) class but of a small group of isolates present in that class. By using the 30% threshold we are selecting only those peaks that are relevant across both the resistant and susceptible classes, therefore the classification is more robust. Tables 1 and 2 in the revised Results section confirm the representativeness of our peaks on both classes and show that none of the peaks selected is present only in a single class.

Bruker Daltonik GmbH. 2011. ClinProTools 3.0: User Manual. Bremen: Bruker Daltonik GmbH

We have edited the Methods section to better explain the 30% spectral peak selection procedure (See Methods- lines 661-668 and below):

Methods – lines 661-668: “Specifically, the peaks were selected if present in at least 30% of all spectra. The 30% threshold was chosen following the workflow suggested in the ClinProTools software documentation (ClinProTools 3.0: User Manual, 2011). In addition, the first pre-processing step of our workflow consists of averaging all the 3 or more technical replicates of each sample. Therefore, after this averaging step we have one spectrum per sample and consequently the 30% threshold used to select the peaks is applied to all samples. By applying the 30% threshold we are selecting only the peaks that are present and hence relevant across both the resistant and susceptible classes, as shown in Tables 1 and 2 in the Results section.”

Bruker Daltonik GmbH. 2011. ClinProTools 3.0: User Manual. Bremen: Bruker Daltonik GmbH

• There is a potential issue in that the data pre-processing was completed on all isolates in one. This may bias the resulting mass spectra and lead to reduced reproducibility of the classification model when used on further validation isolates. Ideally, the authors should complete data pre-processing and statistical mode creation, in my opinion, on a 2/3 training set and then the entire pipeline is validated against the remaining 1/3 of isolates.

The reviewer is correct that by pre-processing all the data together, we could have data leakage problems that could lead to overfitting. However, the reasons behind and in support of our approach are explained below and in the now updated Discussion section lines 485-492.

Although, the Reviewer’s suggestion is correct in terms of avoiding data leakage, the methodological approach we have used to pre-process and apply feature selection mechanism using the whole data is routinely used in the scientific community (Nachtigall *et al*., 2020; Timm, *et al*., 2008; López-Fernández *et al*., 2015; Tong *et al*., 2011; van Oosten and Klein, 2020). In particular, we opted to not pre-process the data using a cross-validation method for two reasons:

(1) The number of resistant samples and their distribution in the minority classes. In particular, the minority classes in our study consist of 15 samples (multidrug-resistant isolates) and 16 samples (benzylpenicillin-resistant isolates). Given the low number of samples in each minority class and that some of the peaks appeared in just a subset of these samples, the split into training and validation sets would have been challenging in terms of number of observations used per set and each partition being enough representative to yield a good peak selection. Moreover, because some of the peaks appeared in just a subset of these samples (minority classes), the random sampling of the data performed during the training model could make these peaks to disappear, and ultimately providing the classifiers with an incorrect set of features. To avoid these problems, we pre-processed all the data together.
(2) The pre-processing methods used in our workflow do not take into account the resistant/susceptible profiles. These profiles are only used later at the machine learning step. The only two pre-processing steps that could be affected by doing a cross-validation with pre-processing would be the peak selection and the standardize methods. However, the first if used in a cross-validation model could increase the chances of getting spurious peaks in the training set that would not represent the whole minority class. While, the second could lead to different mean and standard deviation values between the training and test sets due to the small number of samples in the minority class. All the other pre-processing steps are done using each spectrum individually, therefore there would not be a difference on using a cross validation on them, they would still give the same results.

Discussion – lines 485-492: “In this work, we have opted to pre-process all the data together as previously done by several studies (Nachtigall *et al*., 2020; Timm *et al*., 2008; López-Fernández *et al*., 2015; Tong *et al*., 2011; van Oosten and Klein, 2020) instead of splitting it into a training and validation sets for several reasons. First, given the low number of samples in each of the two minority classes (multidrug resistant and benzylpenicillin-only resistant) it would have been not possible to have a sufficient number of observations in each set and each partition being enough representative to yield a good peak selection. Moreover, because some of the peaks appeared in just a subset of these samples (minority classes), the random sampling of the data could increase the chances of getting spurious peaks in the training set that would not represent the whole minority class. To avoid these problems, we pre-processed all the data together. “

López-Fernández, H., Santos, H.M., Capelo, J.L. *et al.* Mass-Up: an all-in-one open software application for MALDI-TOF mass spectrometry knowledge discovery. BMC Bioinformatics 16, 318 (2015). <https://doi.org/10.1186/s12859-015-0752-4>

Nachtigall, F.M., Pereira, A., Trofymchuk, O.S. *et al.* Detection of SARS-CoV-2 in nasal swabs using MALDI-MS. Nat Biotechnol 38, 1168–1173 (2020). <https://doi.org/10.1038/s41587-020-0644-7>

Timm, W., Scherbart, A., Böcker, S. *et al.* Peak intensity prediction in MALDI-TOF mass spectrometry: A machine learning study to support quantitative proteomics. BMC Bioinformatics 9, 443 (2008). <https://doi.org/10.1186/1471-2105-9-443>

Tong, D.L., Boocock, D.J., Coveney, C. *et al.* A simpler method of preprocessing MALDI-TOF MS data for differential biomarker analysis: stem cell and melanoma cancer studies. Clin Proteom 8, 14 (2011). https://doi.org/10.1186/1559-0275-8-14

van Oosten, Luuk & Klein, Christian. (2020). Machine learning in mass spectrometry: A MALDI-TOF MS approach to phenotypic antibacterial screening. Journal of Medicinal Chemistry. XXXX. 10.1021/acs.jmedchem.0c00040.

• Data availability: the spectra should really be deposited in a public repository according to PLoS Computational Biology guidelines.

All data underlying the findings described will be fully available for research purposes, without restriction, and from the time of publication. PLOS recognizes that, in some instances, authors may not be able to make their underlying data set publicly available for legal or ethical reasons. See the PLoS Computational Biology guidelines (https://journals.plos.org/ploscompbiol/s/data-availability for detailed information and also below. The data used in this project is legally bound to the commercial partner, Quality Milk Management Services (QMMS) that generated it. We will comply to Plos third-data availability policy and guidelines (https://journals.plos.org/ploscompbiol/s/data-availability) regarding third-party data sharing (see below). A Data Availability Statement describing where the data can be found, all necessary contact information, a description of the data set and the third-party source has been included in this submission as requested by the PloS guidelines and will be published in the article, if accepted: Acceptable Data Access Restrictions (https://journals.plos.org/ploscompbiol/s/data-availability)

Plos Computational Biology - Acceptable Data Access Restrictions (https://journals.plos.org/ploscompbiol/s/data-availability)

PLOS recognizes that, in some instances, authors may not be able to make their underlying data set publicly available for legal or ethical reasons. Where these frameworks prevent or limit data release, authors must make these limitations clear in the Data Availability Statement at the time of submission. Acceptable restrictions on public data sharing are detailed below.

Third-party data

For studies involving third-party data. PLOS recognizes, however, that authors may be using third-party data they do not have the rights to share. When third-party data cannot be publicly shared, authors must provide all information necessary for interested researchers to apply to gain access to the data. For any third-party data that the authors cannot legally distribute, they should include the following information in their Data Availability Statement upon submission: A description of the data set and the third-party source. If applicable, verification of permission to use the data set. All necessary contact information others would need to apply to gain access to the data. Authors should properly cite and acknowledge the data source in the manuscript. Please note, if data have been obtained from a third-party source, we require that other researchers would be able to access the data set in the same manner as the authors.

• If cross-validation was performed, then the confidence intervals of the classification models should be reported specifically. Although this is given in Figures 2 and 3, exact values should be written somewhere.

Thanks for the useful suggestion. The Reviewer is right that the exact values should be clearly written in the manuscript. We have updated the Abstract (lines 35-38 and lines 43-45), Author summary (lines 61-65), Results (lines 231-234 and lines 266-269) and Discussion (lines 400-403) with the confidence intervals. The edits have also been reported below:

Abstract – Lines 35-38: ‘For the benzylpenicillin the diagnostic systems achieved up to (mean result ± standard deviation over 30 runs on the test set): accuracy = 97.54% ± 1.91%, sensitivity = 99.93% ± 0.25%, specificity = 95.04% ± 3.83%, and Cohen’s kappa = 95.04% ± 3.83%.’

Abstract – Lines 43-45: ‘Four proteins were able to classify multidrug-resistant and susceptible strains with 96.81% ± 0.43% accuracy. Five proteins, including the previous four, were able to classify benzylpenicillin resistant and susceptible strains with 97.54% ± 1.91% accuracy.’

Author Summary – Lines 61-65: ‘The application to a test set of 82 *S. aureus* isolates collected from 67 cows diagnosed with bovine mastitis across 24 farms discriminated between multidrug-resistant and susceptible strains with (mean result ± standard deviation over 30 runs on the test set) 96.81% ±0.43% accuracy, and between benzylpenicillin-resistant and susceptible strains with 97.54% ± 1.91% accuracy.’

Results – Lines 231-234: ‘Diagnostic systems trained on individual isolates coming from 24 different farms achieved up to (mean result ± standard deviation over 30 runs on the test set): accuracy = 96.81% ±0.43 %, sensitivity = 99.88% ± 0.41%, specificity = 95.96% ± 0.52%, and kappa = 91.83% ± 1.37% in LDA algorithm.’

Results – Lines 266-269: ‘Diagnostic systems trained on individual isolates coming from 24 different farms achieved up to (mean result ± standard deviation over 30 runs on the test set); accuracy = 97.54% ± 1.91%, sensitivity = 99.93% ± 0.25%, specificity = 95.04% ± 3.83%, and kappa = 95.04% ± 3.83% in RBF SVM algorithm.’

Discussion – Lines 400-403: ‘Diagnostic systems trained on individual isolates coming from 24 different farms achieved up to (mean result ± standard deviation over 30 runs on the test set): accuracy = 97.54% ± 1.91%, sensitivity = 99.93% ± 0.25%, specificity = 95.04% ± 3.83%, and kappa = 95.04% ± 3.83% in RBF SVM algorithm.

• There is always a concern with this type of analysis in that they are picking up strain differences rather than susceptibility/resistance differences. Were the isolates strain typed? As two of the four differential proteins were of ribosomal origins, this may be a possibility and an important consideration for the authors to address.

This is a very interesting point. We have not typed the strains. However, to address the Reviewer’s comments and to prove the effectiveness of our method to differentiate susceptibility/resistance profiles rather than strain differences we have:

(i) compared our results to those obtained in studies present in the existing literature focusing on *S. aureus* strain typing.

(ii) carried out further machine learning analyses using the same settings as in the original submission but without the ribosomal proteins as well as with only the ribosomal proteins as input features for classification. The aim of this analysis was to see if in the sole presence of the ribosomal peaks as input features or in their absence the performance of the classifiers changed when compared to the original analysis.

Altogether, considering the results obtained with these new analyses, the comparison of our findings with existing literature together with the findings provided previously in the first submission we believe that it is not unreasonable to assume that we have classified the resistance/susceptibility phenotype and not the strains as explained below. We have updated the Results and Discussion and generated additional supplementary tables and figures to reflect this concept (See Results – lines 292-307; Discussion – lines 521-581, Supplementary Table 3a, Supplementary Table 3b, and below).

(i) Comparison with existing literature works:

We compared the MALDI-TOF spectral peaks (4305.59Da, 4807.21Da, 6422.3Da, 6891.17Da and 9621.26Da) recognised as discriminant by our trained classifiers with the peaks previously found in literature to discriminate the main clonal lineages of *S. aureus* (Wolters *et al*., 2011; Böhme *et al*., 2012; [Camoez *et al*., 2016](https://www.frontiersin.org/articles/10.3389/fmicb.2018.02393/full#B5); Josten *et al*., 2013; Asakura *et al*., 2018). When we compared our peaks with those found by Wolters *et al*. (2011), Böhme *et al*. (2012) and [Camoez *et al*. (2016](https://www.frontiersin.org/articles/10.3389/fmicb.2018.02393/full#B5)) no common peaks were found between the studies. However, similarities were found between our results and the findings reported by Josten *et al*. (2013) and Lasch *et al.* (2014).

In particular, the peaks *m/z* 4305.59 (RpmJ), 6422.3 (RpmD), 6891 (HP2) and 9621.26 (DNA binding protein HU) were revealed to be in common between our study and Josten *et al*. (2013). However, the variant (*m/z* 6397) of the ribosomal protein RpmD found by Josten *et al*. (2013) to be discriminant for the subgroup of CC22 strains was not present in our spectra as we only detected the peak at *m/z* 6422.3 corresponding to RpmD. Moreover, although the protein RpmD was considered a biomarker by Josten *et al*. (2013), it only showed a limited sensitivity (0.167), reflecting a low level of conservation of the mutations in the clonal lineages. For example, the CC22 biomarker was not conserved in all spa types of this clonal complex (Josten *et al*. 2013). The peaks *m/z* 4305.59 (*m/z* 4306 in Josten *et al*. (2013)), 6891 (*m/z* 6889 in Josten *et al*. (2013)) and 9621.26 (*m/z* 9627 in Josten *et al*. (2013)) although identified in the *S. aureus* spectra by Josten *et al*. (2013) were not included in the list of markers distinguishing the different strains. Interestingly, the peaks *m/z* 6889 and 9627 were found the predominant peaks of the *S. aureus* spectrum by Josten *et al*. (2013).

Moreover, Lasch *et al.* (2014) analysed 59 diverse *S. aureus* isolates from 6 different lineages using MALDI-TOF mass spectrometry. Based on their results over a gel view representation and a hierarchical cluster analysis, the authors indicated that, with a few exceptions, CC-specific biomarkers for *S. aureus* are an exception rather than a rule. The authors found 3 regions that could be considered biomarkers for some lineages: *m/z* 3875 and 3891 (CC5); *m/z* 6552 and 6592 (CC8); *m/z* 5002 and 5032 (CC22). Therefore, none of the peaks found in our study were considered biomarkers by Lasch *et al.* (2014). The results found by Lasch *et al.* (2014) clearly suggests that typing *S. aureus* can be rather unsuccessful due to a lack of stable biomarkers to distinct clonal groups, a low classification accuracy based on different CC types and a cluster analysis that indicate the limited possibilities to differentiate *S. aureus* below species levels.

Further comparisons were also made with existing literature coupling MALDI-TOF mass spectrometry with a refined analysis framework to accurately classify resistant and susceptible *S. aureus* strains. In particular, when we compared the peaks (*m/z* 4305.59, 4807.21, 6422.3, 6891.17 and 9621.26) recognised as discriminant for the susceptible and resistant profiles in this study with those previously found (Tang *et al*., 2019; Sogawa *et al*., 2017) differentiating methicillin-susceptible (MSSA) and methicillin-resistant (MRSA) *S. aureus* recovered from clinical samples or at distinguishing VSSA from hVISA/VISA (Wang *et al*., 2018; Mather *et al*., 2016) no similar peaks were detected under the experimental conditions chosen here. In particular, our peaks often mapped in the higher and non-overlapping mass range of the spectrum. Whereas, when we compared our peaks with those found by Asakura *et al*., (2018) to differentiate VISA, hVISA, and VSSA clinical isolates, we found that one peak (*m/z* 4305.59) was in common between the two studies. This peak is among 23 other peaks that were found to be statistically significant among VISA, hVISA and VSSA (p < 10−4, Kruskal-Wallis test). This peak corresponds to the ribosomal protein RpmJ. Indicating that ribosomal proteins can be correlated with resistance phenotypes. This peak corresponds to the ribosomal protein RpmJ, previously also reported by Josten *et al*., (2013) when analysing the peak pattern of 401 MRSA and MSSA strains (see above).

As mentioned in the Introduction and discussion the fact that we found ribosomal proteins can be due to several reasons which may go beyond strain type: In *Staphylococcus spp*., the major targets underlying mechanisms of resistance are the cell envelope, the ribosome and nucleic acids (Foster, 2017). Among those determinants involved in intrinsic resistance, ribosomal proteins have been found to deal with the general response to stress (Olivares Pacheco *et al*., 2013). Similarly, recent findings highlighted the existence of ribosomal mutations conferring resistance to antibiotics of several classes not targeting the ribosome (Gomez *et al*., 2017). Specifically, it has been shown that ribosomal mutations can contribute to the evolution of multidrug resistant profiles, by inducing ribosomal misassemble, that in turn leads to a systematic transcriptional cell alteration, ultimately impacting resistance to multiple antibiotics by interfering with different cellular pathways (Gomez *et al*., 2017) Hence, the ribosomal proteins we identified may possibly represent targets that are indirectly (i.e. stress, beneficial in multidrug environments) related to the resistance phenotype

Ribosomal proteins were also used as biomarkers in the literature (Suarez *et al*., 2013; Fernandez-Alvarez *et al*., 2018, Josten *et al*., 2013) because they are considered housekeeping proteins. Therefore, for some of them it is expected not to show peaks shifts (i.e., mutations) and might not be useful for strain typing (Josten *et al*., 2013).

(ii) Further analytical analyses:

Machine learning analyses were undertaken to see if in the sole presence of the ribosomal peaks as input features or in their absence the performance of the classifiers changed and how this change affected the performance of the classifiers to differentiate susceptibility/resistance profiles. If the ribosomal proteins are in combination with the other proteins found in this work contributing to the resistance/susceptibility phenotype and not to other phenotypes, then, the performance of the classifiers would be slightly decreased when performing the classification with all the other proteins but not the ribosomal proteins.

To prove these hypotheses, we performed two different analyses (see Supplementary Tables 3a and 3b in the revised manuscript and below): (a) using all the non-ribosomal proteins (*m/z* 4807, 6891 and 9621), as input features for the classification and (b) using only the ribosomal proteins (*m/z* 4305 and 6422) as input features. The analysis was done for both the multidrug and benzylpenicillin datasets.

The machine learning results shown below and in Supplementary Table 3 indicate that by removing only the ribosomal proteins from the analysis of both the multidrug and benzylpenicillin datasets, the performance of the classifiers decrease but not significantly, all indicators are still above 80%. However, when using only the ribosomal proteins as input features for the analysis of both multidrug and benzylpenicillin datasets, the specificity and Cohen’s kappa indicators drop to unacceptable values, indicating that the two ribosomal proteins are only important for the susceptible class, but they do not discriminate the resistant samples. Taken all together, these results indicate that the ribosomal proteins are contributing to the susceptibility/resistance results but do not play a major role in the classification. Moreover, these results, together with the literature review done above, indicates that since the peaks selected in this study are not considered important biomarkers for strain typing in *S. aureus,* they should be targeting the classification of resistance aspects only.

Supplementary Table 3a. Supervised machine learning prediction of multidrug resistance spectral signature profiles using the Linear Discriminant Analysis (LDA) classifier. Prediction performance results using all the peaks (*m/z* 4807, 6422, 6891 and 9621); only the non-ribosomal peaks (*m/z* 4807, 6422, 6891 and 9621) and only the ribosomal peak (*m/z* 6422).

|  | **All Proteins** | **Non-ribosomal** | **Ribosomal** |
| --- | --- | --- | --- |
| **Accuracy** | 96.81 ± 0.43 | 93.62 ± 1.55 | 77.25 ± 0.00 |
| **Sensitivity** | 99.88 ± 0.41 | 96.07 ± 0.10 | 100.00 ± 0.00 |
| **Specificity** | 95.96 ± 0.52 | 85.33 ± 6.75 | 0.00 ± 0.00 |
| **Cohen's kappa** | 91.83 ± 1.37 | 81.00 ± 5.85 | 0.00 ± 0.00 |

Supplementary Table 3b. Supervised machine learning prediction of multidrug resistance spectral signature profiles using a non-linear (RBF kernel) support vector machine (RBF-SVM) classifier. Prediction performance results using all the peaks (*m/z* 4305, 4807, 6422, 6891 and 9621); only the non-ribosomal peaks (*m/z* 4807, 6422, 6891 and 9621) and only the ribosomal peak (*m/z* 4305 and 6422).

|  | **All Proteins** | **Non-ribosomal** | **Ribosomal** |
| --- | --- | --- | --- |
| **Accuracy** | 97.54 ± 1.91 | 93.14 ± 1.22 | 75.99 ± 1.86 |
| **Sensitivity** | 99.93 ± 0.25 | 95.24 ± 1.18 | 98.24 ± 2.37 |
| **Specificity** | 95.04 ± 3.83 | 86.00 ± 3.59 | 0.44 ± 1.66 |
| **Cohen's kappa** | 95.04 ± 3.83 | 80.38 ± 3.54 | -1.54 ± 3.22 |

These results can also be explained by the fact that our study analysed 82 isolates, collected over a 1-year period from 24 farms spread in England and Wales. It is not unreasonable to assume that the isolates included in this study were different. Our supervised learning-based classifier consisted of a binary classification (resistant/susceptible), where each observation (isolate) was labelled according to the MIC values obtained for each specific isolate. Given the high performance indicators accompanying the classification and given the variety of different peaks among strains as shown by Josten *et al*. (2013), Wolters *et al*. (2011), Böhme *et al*. (2012) and [Camoez *et al*. (2016](https://www.frontiersin.org/articles/10.3389/fmicb.2018.02393/full#B5)), it is very unlikely that we could separate all the different strains circulating in just two groups and importantly with such high performance indicators. From a machine learning point of view, given the limited number of observations, relative high number of possible strains, a binary outcome, number of genetic/molecular traits different among the strains it would not had been possible to separate the different strains in just two groups especially with such high-performance scores. This is also in agreement with Lasch et al (2014) that although performing an elegant modular/hierarchical ANN analysis of spectra from the *S. aureus* data set (we only did a one-step machine learning classification), apart from a fairly good classification accuracy for strains of *S. aureus* CC8 (91%) and, to a lesser extent for strains of CC5 (80%) and CC30 (78%), the classification accuracy for CC398 (69%), CC22 (43%) and CC45 (40%) strains was unacceptably low. Despite intensive efforts aiming at improving these outcomes, neither variations of the spectral pre-processing (vide infra) nor of the network topology resulted in better classification results.

We have now edited the manuscript see Results line 292-307, Discussion lines 521-581 and below:

See Results– lines 292-307:

“**Machine learning analyses undertaken to prove the effectiveness of our method to differentiate susceptibility/resistance profiles rather than strain differences**

Because two of the five discriminant proteins found in this work were of ribosomal origins and ribosomal proteins have been used for the discrimination of major *S. aureus* lineages based on MALDI-TOF analysis (Josten *et al*., 2013; Wolters *et al*., 2011; Böhme *et al*., 2012; and [Camoez *et al*., 2016](https://www.frontiersin.org/articles/10.3389/fmicb.2018.02393/full#B5)) we performed further analyses in support that our classifiers were picking up susceptibility/resistance differences rather than strain differences. First, we investigated if and how in the sole presence of the ribosomal peaks as input features or in their absence the performance of the classifiers changed and how. As shown in Supplementary Table 3 by removing only the ribosomal proteins from the analysis of both multidrug and benzylpenicillin datasets, the performance of the classifiers decreases but not significantly, all indicators are still above 80%. However, when using only the ribosomal proteins as input features for the analysis of both multidrug and benzylpenicillin datasets, the specificity and Cohen’s kappa indicators drop to unacceptable values for both the multidrug and benzylpenicillin predicted phenotypes. Altogether these results indicate that the ribosomal proteins in combination with the other discriminant proteins are contributing to the susceptibility/resistance classification but do not play a major role in the classification. “

See Discussion – lines 521-581: “In addition to the machine learning analyses undertaken to prove the effectiveness of our method to differentiate susceptibility/resistance profiles rather than strain differences, we also compared the MALDI-TOF spectral peaks (4305.59Da, 4807.21Da, 6422.3Da, 6891.17Da and 9621.26Da) recognised as discriminant by our trained classifiers with the peaks previously found in literature to discriminate the main clonal lineages of *S. aureus* (Wolters *et al*., 2011; Böhme *et al*., 2012; [Camoez *et al*., 2016](https://www.frontiersin.org/articles/10.3389/fmicb.2018.02393/full#B5); Josten *et al*., 2013, Asakura *et al*., 2018; Lasch *et al*., 2014). When we compared our peaks with those found by Wolters *et al*. (2011), Böhme *et al*. (2012) and [Camoez *et al*. (2016](https://www.frontiersin.org/articles/10.3389/fmicb.2018.02393/full#B5)) no common peaks were found between the studies. However, similarities were found between our results and the findings reported by Josten *et al*. (2013) and Lasch *et al*. (2014).

In particular, the peaks *m/z* 4305.59 (RpmJ), 6422.3 (RpmD), 6891 (HP2) and 9621.26 (DNA binding protein HU) were revealed to be in common between our study and Josten *et al*. (2013). However, the variant (*m/z* 6397) of the ribosomal protein RpmD found by Josten *et al*. (2013) to be discriminant for the subgroup of CC22 strains was not present in our spectra as we only detected the peak at *m/z* 6422.3 corresponding to RpmD. Moreover, although the protein RpmD was considered a biomarker by Josten *et al*. (2013), it only showed a limited sensitivity (0.167), reflecting a low level of conservation of the mutations in the clonal lineages. For example, the CC22 biomarker was not conserved in all spa types of this clonal complex (Josten *et al*. 2013). The peaks *m/z* 4305.59 (*m/z* 4306 in Josten *et al*. (2013)), 6891 (*m/z* 6889 in Josten *et al*. (2013)) and 9621.26 (*m/z* 9627 in Josten *et al*. (2013)) although identified in the *S. aureus* spectra by Josten *et al*. (2013) were not included in the list of markers distinguishing the different strains. Moreover, Lasch *et al.* (2014) analysed 59 diverse *S. aureus* isolates from 6 different lineages using MALDI-TOF mass spectrometry. Based on their results over a gel view representation and a hierarchical cluster analysis, the authors indicated that, with a few exceptions, CC-specific biomarkers for *S. aureus* are an exception rather than a rule. The authors found 3 regions that could be considered biomarkers for some lineages: *m/z* 3875 and 3891 (CC5); *m/z* 6552 and 6592 (CC8); *m/z* 5002 and 5032 (CC22). Therefore, none of the peaks found in our study were considered biomarkers by Lasch *et al*. (2014). The results found by Lasch *et al*. (2014) clearly suggests that typing *S. aureus* can be rather unsuccessful due to a lack of stable biomarkers to distinct clonal groups, a low classification accuracy based on different CC types and a cluster analysis that indicate the limited possibilities to differentiate *S. aureus* below species levels.

Further comparisons were also made with existing literature coupling MALDI-TOF mass spectrometry with a refined analysis framework to accurately classify resistant and susceptible *S. aureus* strains. In particular, the peaks (*m/z* 4305.59, 4807.21, 6422.3, 6891.17 and 9621.26) recognised as discriminant for the susceptible and resistant profiles in this study with those previously found (Tang *et al*., 2019; Sogawa *et al*., 2017) differentiating MSSA and MRSA recovered from clinical samples or at distinguishing VSSA from hVISA/VISA (Wang *et al*., 2018; Mather *et al*., 2016) no similar peaks were detected under the experimental conditions chosen here. In particular, our peaks often mapped in the higher and non-overlapping mass range of the spectrum. Whereas, when we compared our peaks with those found by Asakura *et al*., (2018) to differentiate VISA, hVISA, and VSSA clinical isolates, we found that one peak (*m/z* 4305.59) was in common between the two studies. This peak is among 23 other peaks that were found to be statistically significant among VISA, hVISA and VSSA (p < 10−4, Kruskal-Wallis test). This peak also corresponds to the ribosomal protein RpmJ. Indicating that ribosomal proteins can be correlated with resistance phenotypes. This was also reported by Josten *et al*., (2013) when analysing the peak pattern of 401 MRSA and MSSA strains (see above).

Although we have not typed our strains, which we acknowledge as a limitation of our study, we believe that it is not unreasonable to assume that we have classified the resistance/susceptibility phenotype and not the strains. Our supervised learning-based classifier consisted of a binary classification (resistant/susceptible), where each observation (isolate) was labelled according to the MIC values obtained for each specific isolate. Given the high performance indicators accompanying our classification results and given the variety of different peaks among strains as shown by Josten *et al*. (2013), Wolters *et al*. (2011), Böhme *et al*. (2012) and [Camoez *et al*. (2016](https://www.frontiersin.org/articles/10.3389/fmicb.2018.02393/full#B5)) Lasch *et al.* (2014), it is very unlikely that we could separate all the different strains circulating in just two groups and importantly with such high-performance indicators. From a machine learning point of view, given the limited number of observations, relative high number of possible strains, binary outcome, number of genetic/molecular traits different among the strains it would not had been possible to separate the different strains in just two groups especially with such high-performance scores. This is also in agreement with Lasch *et al.* (2014) that although performing an elegant modular/hierarchical ANN analysis of spectra from the *S. aureus* data set (we only did a one-step machine learning classification), apart from a fairly good classification accuracy for CC8 strains of *S. aureus* and, to a lesser extent for strains of CC5 (80%) and CC30 (78%), the classification accuracy for the other strains was unacceptably low. Despite intensive efforts aiming at improving these outcomes, neither variations of the spectral pre-processing nor of the network topology resulted in better classification results according to the authors.”

Asakura, K., Azechi, T., Sasano, H., Matsui, H., Hanaki, H., Miyazaki, M., Takata, T., Sekine, M., Takaku, T., Ochiai, T., Komatsu, N., Shibayama, K., Katayama, Y., & Yahara, K. (2018). Rapid and easy detection of low-level resistance to vancomycin in methicillin-resistant Staphylococcus aureus by matrix-assisted laser desorption ionization time-of-flight mass spectrometry. PloS one, 13(3), e0194212. https://doi.org/10.1371/journal.pone.0194212

Böhme K, Morandi S, Cremonesi P, Fernández No IC, Barros-Velázquez J, Castiglioni B, Brasca M, Cañas B, Calo-Mata P. Characterization of Staphylococcus aureus strains isolated from Italian dairy products by MALDI-TOF mass fingerprinting. Electrophoresis. 2012 Aug;33(15):2355-64. doi: 10.1002/elps.201100480. PMID: 22887156.

Camoez, M., Sierra, J. M., Dominguez, M. A., Ferrer-Navarro, M., Vila, J., and Roca, I. (2016). Automated categorization of methicillin-resistant Staphylococcus aureus clinical isolates into different clonal complexes by MALDI-TOF mass spectrometry. Clin. Microbiol. Infect. 22, 161 e1–e7. doi: 10.1016/j.cmi.2015.10.009

Christiansen, M. T. (2014). Characterization of methicillin-resistant Staphylococcus aureus Sequence Type 398. National Food Institute, Technical University of Denmark.

Foster TJ. Antibiotic resistance in Staphylococcus aureus. Current status and future prospects. FEMS Microbiology Reviews. 2017;41(3):430-49.

Gomez JE, Kaufmann-Malaga BB, Wivagg CN, Kim PB, Silvis MR, 625 Renedo N, *et al.* Ribosomal mutations promote the evolution of antibiotic resistance in a multidrug environment. eLife. 627 2017;6:e20420.

Josten, M., Reif, M., Szekat, C., Al-Sabti, N., Roemer, T., Sparbier, K., Kostrzewa, M., Rohde, H., Sahl, H. G., & Bierbaum, G. (2013). Analysis of the matrix-assisted laser desorption ionization-time of flight mass spectrum of Staphylococcus aureus identifies mutations that allow differentiation of the main clonal lineages. Journal of clinical microbiology, 51(6), 1809–1817. <https://doi.org/10.1128/JCM.00518-13>

Lasch P, Fleige C, Stämmler M, Layer F, Nübel U, Witte W, Werner G. Insufficient discriminatory power of MALDI-TOF mass spectrometry for typing of Enterococcus faecium and Staphylococcus aureus isolates. J Microbiol Methods. 2014 May;100:58-69. doi: 10.1016/j.mimet.2014.02.015. Epub 2014 Mar 12.

Mather, C. A., Werth, B. J., Sivagnanam, S., SenGupta, D. J., and Butler-Wu, S. M. (2016). Rapid detection of vancomycin-intermediate Staphylococcus aureus by matrix-assisted laser desorption ionization-time of flight mass spectrometry. J. Clin. Microbiol. 54, 883–890. doi: 10.1128/JCM.02428-15

Olivares Pacheco J, Bernardini A, Garcia-Leon G, Corona F, Sanchez MB, Martinez J. The intrinsic resistome of bacterial pathogens. Frontiers in Microbiology. 2013;4:103

Sogawa K, Watanabe M, Ishige T, Segawa S, Miyabe A, Murata S, Saito T, Sanda A, Furuhata K, Nomura F. Rapid Discrimination between Methicillin-Sensitive and Methicillin-Resistant Staphylococcus aureus Using MALDI-TOF Mass Spectrometry. Biocontrol Sci. 2017;22(3):163-169. doi: 10.4265/bio.22.163. PMID: 28954959.

Tang W, Ranganathan N, Shahrezaei V, Larrouy-Maumus G (2019) MALDI-TOF mass spectrometry on intact bacteria combined with a refined analysis framework allows accurate classification of MSSA and MRSA. PLOS ONE 14(6):e0218951. <https://doi.org/10.1371/journal.pone.0218951>

Wolters, M., Rohde, H., Maier, T., Belmar-Campos, C., Franke, G., Scherpe, S., *et al.* (2011). MALDI-TOF MS fingerprinting allows for discrimination of major methicillin-resistant Staphylococcus aureus lineages. Int. J. Med. Microbiol. 301, 64–68. doi: 10.1016/j.ijmm.2010.06.002

• It was also be good to show a breakdown of isolates by susceptibility/resistance by farm location. Were they evenly distributed amongst farms or was the model driven by differences in location of collection?

We have now updated Figure 1 to show a breakdown of isolates by susceptibility/resistance by farm location. We have also generated an additional Supplementary Table 1 reporting the breakdown of isolates by susceptibility/resistance by farm location. The farms differed in terms of number of susceptible /resistant cases. It was our intention to use samples reflecting the real situation in the farms and not to generate an equally distributed sample collection. This is because, only by analysing real cases we will be able to develop predictive models that can be effective in analysing the variability present in the farms. Working with farms differing in terms of number of susceptible /resistant cases will force the classifier to leverage differences among farms and to develop more robust models that may have higher potential to be applied at a larger scale.

• Was there any correlation between MIC levels and protein intensities?

We could not find any linear correlation between the MIC levels and the proteins intensities; this is why we opted for a more sophisticated and powerful analysis such as machine learning to identify correlative patterns in the data. However, to address the comment posed by the Reviewer and show the absence of correlation we have created a pair plot for the benzylpenicillin only vs susceptible data set, describing the Pearson correlation between all combinations of two peaks for the resistant and susceptible samples (see plot below). In the plot is also possible to analyse the distribution of each individual peak and compare with the MIC values.

The plot below indicates that for three peaks (4.807kDa, 6.891kDa and 9.621kDa) the mean intensity is higher in the susceptible samples than in the resistant ones which could indicate a negative correlation, since the MIC values for susceptible samples is smaller when compared to the resistant ones. For the other two peaks, the mean intensity is the same between resistant and susceptible samples.

Figure: Pair plot pairwise relationship between the peaks found on the benzylpenicillin vs susceptible data set. The resistant and susceptible intensities are coloured red and blue, respectively. The plots below the diagonal show the relationship between two peaks for resistant and susceptible classes; this relationship is in terms of the normalized intensity. The diagonal shows the distribution of the normalized intensity of each peak; the y-axis indicates the frequency while the x-axis indicates the normalized intensity. The values above the diagonal shows the Person correlation for the relationship between two peaks for resistant and susceptible classes.

• As for earlier comment, this is not the first use of MALDI-TOF for in antibiotic susceptibility profiling in *S. aureus*. Did previous studies, albeit using *S. aureus* isolated from humans or non-bovine sources show the same proteins as differential?

This is again an interesting question that undoubtedly helped us to improve the manuscript. We have updated the discussion by making relevant comparisons with other studies/peaks that were missing indeed. (See Discussion – lines 549-562 and below)

Discussion – lines 549-562: “Further comparisons were also made with existing literature coupling MALDI-TOF mass spectrometry with a refined analysis framework to accurately classify resistant and susceptible S. aureus strains. In particular, the peaks (m/z 4305.59, 4807.21, 6422.3, 6891.17 and 9621.26) recognised as discriminant for the susceptible and resistant profiles in this study with those previously found (Tang et al., 2019; Sogawa et al., 2017) differentiating MSSA and MRSA recovered from clinical samples or at distinguishing VSSA from hVISA/VISA (Wang et al., 2018; Mather et al., 2016) no similar peaks were detected under the experimental conditions chosen here. In particular, our peaks often mapped in the higher and non-overlapping mass range of the spectrum. Whereas, when we compared our peaks with those found by Asakura et al., (2018) to differentiate VISA, hVISA, and VSSA clinical isolates, we found that one peak (m/z 4305.59) was in common between the two studies. This peak is among 23 other peaks that were found to be statistically significant among VISA, hVISA and VSSA (p < 10−4, Kruskal-Wallis test). This peak also corresponds to the ribosomal protein RpmJ. Indicating that ribosomal proteins can be correlated with resistance phenotypes. This was also reported by Josten et al., (2013) when analysing the peak pattern of 401 MRSA and MSSA strains (see above).”

Tang W, Ranganathan N, Shahrezaei V, Larrouy-Maumus G (2019) MALDI-TOF mass spectrometry on intact bacteria combined with a refined analysis framework allows accurate classification of MSSA and MRSA. PLOS ONE 14(6):e0218951. <https://doi.org/10.1371/journal.pone.0218951>

Asakura K, Azechi T, Sasano H, Matsui H, Hanaki H, *et al.* (2018) Rapid and easy detection of low-level resistance to vancomycin in methicillin-resistant Staphylococcus aureus by matrix-assisted laser desorption ionization time-of-flight mass spectrometry. PLOS ONE 13(3): e0194212. <https://doi.org/10.1371/journal.pone.0194212>

Mather, C. A., Werth, B. J., Sivagnanam, S., SenGupta, D. J., and Butler-Wu, S. M. (2016). Rapid detection of vancomycin-intermediate Staphylococcus aureus by matrix-assisted laser desorption ionization-time of flight mass spectrometry. J. Clin. Microbiol. 54, 883–890. doi: 10.1128/JCM.02428-15

Sogawa K, Watanabe M, Ishige T, Segawa S, Miyabe A, Murata S, Saito T, Sanda A, Furuhata K, Nomura F. Rapid Discrimination between Methicillin-Sensitive and Methicillin-Resistant Staphylococcus aureus Using MALDI-TOF Mass Spectrometry. Biocontrol Sci. 2017;22(3):163-169. doi: 10.4265/bio.22.163. PMID: 28954959.

Wang HY, Chen CH, Lee TY, Horng JT, Liu TP, Tseng YJ, Lu JJ. Rapid Detection of Heterogeneous Vancomycin-Intermediate Staphylococcus aureus Based on Matrix-Assisted Laser Desorption Ionization Time-of-Flight: Using a Machine Learning Approach and Unbiased Validation. Front Microbiol. 2018 Oct 11;9:2393. doi: 10.3389/fmicb.2018.02393.

**Reviewer #2**

**General comments:**

The work aims to develop a MALDI-TOF-based approach to discriminate between antibiotic-resistant and -susceptible *S. aureus* isolates recovered from bovine mastitis. Since this approach is faster and simpler than conventional ways for determining antimicrobial susceptibility, it represents a potential alternative that could be useful in the veterinary field. However, few points should be addressed throughout the manuscript, as follows:

- In the Introduction, the authors mention the emergence of penicillinase-producing *S. aureus* strains back in the 50's. I believe it should be also mentioned in the Introduction that production of alternative PBPs (coded by mecA for example) are nowadays more important than penicillinase production.

Thanks for the useful suggestion. The Introduction has now been updated (see Introduction line 97-112 and below)

Introduction – Line 97-112: ‘In 1959 a penicillin derivative, methicillin, that was resistant to β-lactamase hydrolysis was synthetized. However, immediately after methicillin was used clinically, methicillin-resistant *S. aureus* (MRSA) strains were isolated (Chambers, 1997; Stapleton & Taylor, 2002). Resistance to methicillin is conferred by the acquisition of a mobile genetic element, the staphylococcal cassette chromosome (SCCmec) carrying the gene *mecA* encoding a penicillin-binding protein (PBP2a) (Chambers, 1997; Stapleton & Taylor, 2002). Over the years, mutations, acquisition and accumulation of antibiotic resistance-conferring genes, divergent *mecA* gene homologues (*mecC*) (Paterson *et al*., 2014; García-Álvarez *et al*., 2011) and SCCmec elements (Paterson *et al*., 2014) have led to the emergence of multi-resistant MRSA strains (Livermore, 2000).

Nowadays, MRSA are resistant to virtually all β-lactam antibiotics (Paterson *et al.*, 2014). Since its emergence in the early 2000’s, livestock-associated methicillin-resistant *S. aureus* (LA-MRSA) has become an emerging problem in many parts of the world (Fluit, 2012; Anjum *et al*., 2019; Smith, 2015). The detection of *mecC* MRSA from dairy cattle in England (Garcia-Alvarez *et al.*, 2011) was reported in 2011. The first isolation of both *mecA* and *mecC* LA-MRSA. In bulk milk from dairy cattle in the UK was reported in 2012 (Paterson *et al*., 2012). Worryingly, a number of studies have suggested of possible human-livestock MRSA transmissions (Smith, 2015; Spoor *et al*., 2013; Wu *et al*., 1996; Garcia-Alvarez *et al*., 2011; Shore *et al*., 2011). In addition, several studies have reported that persons with occupational livestock exposure may be at increased risk of becoming colonized with LA-MRSA (Goerge *et al*., 2017).

Anjum, M. F., Marco-Jimenez, F., Duncan, D., Marín, C., Smith, R. P., & Evans, S. J. (2019). Livestock-Associated Methicillin-Resistant Staphylococcus aureus From Animals and Animal Products in the UK. Frontiers in microbiology, 10, 2136. <https://doi.org/10.3389/fmicb.2019.02136>

Chambers HF. Methicillin resistance in staphylococci: molecular and biochemical basis and clinical implications. Clin Microbiol Rev. 1997 Oct;10(4):781-91. doi: 10.1128/CMR.10.4.781-791.1997. PMID: 9336672; PMCID: PMC172944.

Fluit AC. Livestock-associated Staphylococcus aureus. Clin Microbiol Infect. 2012 Aug;18(8):735-44. doi: 10.1111/j.1469-0691.2012.03846.x. Epub 2012 Apr 18. PMID: 22512702.

Garcia-Alvarez L, Holden MT, Lindsay H, Webb CR, Brown DF, *et al.* (2011) Meticillin-resistant Staphylococcus aureus with a novel mecA homologue in human and bovine populations in the UK and Denmark: a descriptive study. Lancet Infect Dis 11: 595–603. pmid:21641281Livermore DM. Antibiotic resistance in staphylococci. Int J Antimicrob Agents. 2000 Nov;16 Suppl 1:S3-10. doi: 10.1016/s0924-8579(00)00299-5. PMID: 11137402.

Goerge T., Lorenz M. B., van Alen S., Hubner N. O., Becker K., Kock R. (2017). MRSA colonization and infection among persons with occupational livestock exposure in Europe: prevalence, preventive options and evidence. Vet. Microbiol. 200 6–12. 10.1016/j.vetmic.2015.10.027

Livermore DM. Antibiotic resistance in staphylococci. Int J Antimicrob Agents. 2000 Nov;16 Suppl 1:S3-10. doi: 10.1016/s0924-8579(00)00299-5. PMID: 11137402.

Paterson, G. K., Harrison, E. M., & Holmes, M. A. (2014). The emergence of mecC methicillin-resistant Staphylococcus aureus. Trends in microbiology, 22(1), 42–47. <https://doi.org/10.1016/j.tim.2013.11.003>

Shore AC, Deasy EC, Slickers P, Brennan G, O’Connell B, *et al.* (2011) Detection of staphylococcal cassette chromosome mec type XI carrying highly divergent mecA, mecI, mecR1, blaZ, and ccr genes in human clinical isolates of clonal complex 130 methicillin-resistant Staphylococcus aureus. Antimicrob Agents Chemother 55: 3765–3773. pmid:21636525

Smith TC (2015) Livestock-Associated Staphylococcus aureus: The United States Experience. PLOS Pathogens 11(2): e1004564. <https://doi.org/10.1371/journal.ppat.1004564>

Spoor LE, McAdam PR, Weinert LA, Rambaut A, Hasman H, *et al.* (2013) Livestock origin for a human pandemic clone of community-associated methicillin-resistant Staphylococcus aureus. MBio 4: e00356–13. pmid:23943757

Stapleton, P. D., & Taylor, P. W. (2002). Methicillin resistance in Staphylococcus aureus: mechanisms and modulation. Science progress, 85(Pt 1), 57–72. https://doi.org/10.3184/003685002783238870

Wu S, Piscitelli C, de Lencastre H, Tomasz A (1996) Tracking the evolutionary origin of the methicillin resistance gene: cloning and sequencing of a homologue of mecA from a methicillin susceptible strain of Staphylococcus sciuri. Microb Drug Resist 2: 435–441. pmid:9158816

- Throughout the manuscript revise the term "multidrug-resistance" for standardization purposes, as in certain places it is written "multi-drug resistance" (including in the title of the manuscript).

We have corrected the term "multidrug-resistance" throughout the manuscript

**-** Were the 82 *S. aureus* isolates of the study chosen among a larger collection of isolates? If so, which were the selection criteria?

The Reviewer is correct, our criteria for selecting the isolates to be used was the quality of the spectra. At least three good quality replicates were required for inclusion of the isolate in the analysis. However, all the isolates used in this study had more than 3 good quality replicates, so no isolates were removed because of this selection criterion. Moreover, when three qualifying technical replicates could not be obtained the sample was re-analysed in order to get at least 3 replicates. Therefore, in this study all isolates (n = 82) collected for the study were used as they all met the quality criteria. We have now updated the Method section to better clarify the collection/selection issue (See Method, lines 632-635 and Below). In addition, we have edited Figure 1 to highlight the breakdown of the 82 isolates collection by susceptibility/resistance by farm location.

Method - Line 632-635: “At least three good quality spectra per isolate were required for inclusion of the isolate in the analysis. Moreover, when three qualifying technical replicates could not be obtained the sample was re-analysed in order to get at least 3 replicates. All the 82 isolates used in this study had three good quality technical replicates”

Also, wouldn't 82 isolates be a small number of samples to be used as a test collection for a new methodology?

As correctly pointed out by the reviewer this study has been confined to a relatively small number of isolates. Ideally a larger number of isolates would have allowed to refine the machine learning predictions. However, we believe that our models received sufficient training and the generated models are sufficiently robust as proved by the performance indicators. We have edited the discussion to better explain this concept. In particular, we have acknowledged the limitations of our approach due the small sample size and explained the reasons underlying the robustness of the approach although such limitation. Please see Discussion lines 494-520 and below.

Discussion – lines 494-520: “Moreover, this study has been confined to a relatively small number of isolates. Ideally, a larger number of samples would have allowed to refine the machine learning predictions. However, other studies attempted the analysis of antimicrobial resistance on *S. aureus* with MALDI-TOF and machine learning and a similar sample size. For example, Tang *et al*. (2019), to implement hVISA detection models, examined 10 methicillin-susceptible *S. aureus* (MSSA) and 10 MRSA clinical isolates recovered from individual patients. Wang *et al*. (2018), used MALDI-TOF mass spectra obtained from 35 hVISA/ VISA and 90 VSSA isolates. Mather *et al*. (2016), tested 21 VISA, 21 hVISA, and 38 VSSA isolates to develop their SVM based models. Usually, the larger the dataset the greater is the statistical power for pattern recognition. However, in our machine learning approach we have used the Nested CV approach which is known to produce robust and unbiased performance estimates regardless of sample size (Vabalas *et al*., 2019). The machine learning performance indicators associated with our models are high suggesting that models were sufficiently trained.

In addition, we acknowledge, as a limitation of this study, that our data were collected from farms only in England and Wales. However, this should not pose a restriction on our method’s ability to predict resistance or susceptibility in other farms across the globe. If it is given a sufficiently diverse distribution of data to train the supervised learning algorithms, this would reduce any geographical bias that could affect predictive capability. This study should be considered a proof-of-principle where we conducted a feasibility work to invest on with larger samples and geographical areas. Finally, the downside of requiring larger sample sizes is limitations in data availability, often requiring reliance on public databases and thus compromise on the type of available data and possible studies. Unfortunately, in omics and in other technology-based data collection analysis, very often only small samples are available, this is because of limited in vivo experiments, protocols, involvement of human participants and costs. For example, whilst not being able to rely on large amounts of data, we had the unprecedented possibility to demonstrate that our methodology is associated with high classification accuracy even when using small sample size, this applicability may facilitate research scenarios where only limited data is available.”

Mather CA *et al.*, Rapid Detection of Vancomycin-Intermediate Staphylococcus aureus by Matrix-Assisted Laser Desorption Ionization–Time of Flight Mass Spectrometry. J Clin Microbiol. 2016; 54(4):883-90

Tang W *et al.*, MALDI-TOF mass spectrometry on intact bacteria combined with a refined analysis framework allows accurate classification of MSSA and MRSA. PLoS One. 2019 ;14(6):e0218951.

Vabalas A *et al.*, Machine learning algorithm validation with a limited sample size. PLoS One. 2019; 14(11): e0224365.

Wang HY, Chen CH, Lee TY, Horng JT, Liu TP, Tseng YJ, Lu JJ. Rapid Detection of Heterogeneous Vancomycin-Intermediate Staphylococcus aureus Based on Matrix-Assisted Laser Desorption Ionization Time-of-Flight: Using a Machine Learning Approach and Unbiased Validation. Front Microbiol. 2018 Oct 11;9:2393. doi: 10.3389/fmicb.2018.02393.

- Usually, classification of bacteria as multidrug resistant requires that they show resistance to at least three different classes of antibiotics. That is quite a common sense in the literature. Why in the study the authors considered multidrug resistance when isolates were resistant to two antibiotics? Is there any reference that supports this?

As correctly pointed out by the reviewer the definition for multidrug resistant (MDR) is standardized within the human healthcare where MDR bacteria were defined as bacteria that are non-susceptible to at least one antimicrobial agent in three or more antimicrobial classes. However, in veterinary medicine there are still no accepted definitions and numerous reports have used different terminology and assessments of MDR (Sweeney *et al*., 2018).

However, as correctly pointed out by the reviewer, in the original manuscript we have indeed given a misleading definition of our samples. In particular, our cohort consist of resistant isolates, among these, 16 isolates were found to be only penicillin-resistant, while 15 isolates had resistance to multiple antibiotics. Among these 15 isolates, 13 were found to be resistant to 3 or more antibiotics, with at least one antimicrobial agent in three antimicrobial classes (multidrug-resistant), while two isolates were resistant to 2 or more antibiotics with at least one antimicrobial agent in two antimicrobial classes (extensively drug-resistant, XDR). Specifically, out of 15 multidrug-resistant isolates, 11 isolates were resistant to benzylpenicillin, clindamycin, erythromycin, tilmicosin and tylosin; 1 isolate was resistant to benzylpenicillin, clindamycin, tilmicosin and tylosin; 1 isolate was resistant to benzylpenicillin, tetracycline and tilmicosin; 1 isolate was resistant to benzylpenicillin and tetracycline, and 1 isolate was resistant to benzylpenicillin, cefalotin, cefoxitin and oxacillin. Please see below for the breakdown of isolates, antibiotics and antimicrobial classes in our cohort. The majority of our isolates meet the multidrug definition used in human medicine, except for two of them that are XDR. We have now updated the Results and Discussion (See Results – lines 184-194 and lines 236-237, Discussion 513-517 and Figure 2 see also below) to better explain the multidrug resistance annotation in our cohort. We’d rather keep the multidrug definition, given the majority of the MDR samples and for simplicity, but adding the reviewer suggestion about clarifying this (MDR and XDR) properly in the manuscript to avoid any misunderstandings.

The breakdown of the samples is the following:

| **Antibiotic** | **Class** | **Count** |
| --- | --- | --- |
| benzylpenicillin | Penicillin | 11 isolates |
| Clindamycin | Lincosamide |  |
| erythromycin | Macrolides |  |
| Tilmicosin | Macrolides |  |
| Tylosin | Macrolides |  |

| **Antibiotic** | **Class** | **Count** |
| --- | --- | --- |
| benzylpenicillin | Penicillin | 1  isolates |
| Clindamycin | Lincosamide |  |
| Tilmicosin | Macrolides |  |
| Tylosin | Macrolides |  |

| **Antibiotic** | **Class** | **Count** |
| --- | --- | --- |
| benzylpenicillin | Penicillin | 1  isolates |
| Tetracycline | Tetracycline |  |
| Tilmicosin | Macrolides |  |

| **Antibiotic** | **Class** | **Count** |
| --- | --- | --- |
| benzylpenicillin | Penicillin | 1  isolates |
| Tetracycline | Tetracycline |  |

| **Antibiotic** | **Class** | **Count** |
| --- | --- | --- |
| benzylpenicillin | Penicillin | 1  isolates |
| Cefalotin | Cepahlosporin |  |
| Cefoxitin | Cepahlosporin |  |
| Oxacillin | Penicillin |  |

The Results, Discussion and Methods have been updated (See Results – lines 170-180 and lines 217-218, Discussion 481-482, Figure 2 and below)

Results - line 170-180 “Amongst the resistant isolates, 16 isolates were found to be only penicillin-resistant, while 15 isolates had resistance to multiple antibiotics, among these 15 isolates 13 were found to be resistant to three or more antibiotics, with at least one antimicrobial agent in three antimicrobial classes (multidrug-resistant, MDR), while two isolates were resistant to two or more antibiotics with at least one antimicrobial agent in two antimicrobial classes (extensively drug-resistant, XDR). We considered the MDR and XDR as one class and named it as MDR for simplicity. As shown in Figure 2, out of 15 multidrug-resistant isolates, 11 isolates were resistant to benzylpenicillin, clindamycin, erythromycin, tilmicosin and tylosin; 1 isolate was resistant to benzylpenicillin, clindamycin, tilmicosin and tylosin; 1 isolate was resistant to benzylpenicillin, tetracycline and tilmicosin; 1 isolate was resistant to benzylpenicillin and tetracycline, and 1 isolate was resistant to benzylpenicillin, cefalotin, cefoxitin and oxacillin.”

Results – lines 217-218: Specifically, we considered the spectra of 15 multidrug-resistant isolates (13 MDR and 2 XDR).

Discussion – lines 481-482: The MDR and XDR isolates, collectively named multidrug-resistant isolates, used in this study were all resistant to benzylpenicillin in addition to other antimicrobial agents.

Sweeney MT, Lubbers BV, Schwarz S, Watts JL. Applying definitions for multidrug resistance, extensive drug resistance and pandrug resistance to clinically significant livestock and companion animal bacterial pathogens. J Antimicrob Chemother. 2018 Jun 1;73(6):1460-1463. doi: 10.1093/jac/dky043.

- One result stands out: none of the *S. aureus* isolates was resistant to cefoxitin or oxacillin, despite being resistant to penicillin. For human isolates of *S. aureus*, initial screening and detection of MRSA is usually based on the in vitro susceptibility to cefoxitin or oxacillin. In this regard, one could suggest that penicillin-resistant *S. aureus* isolates in this study were maybe indeed producers of penicillinase instead of being MRSA. This could be included in the discussion, or *S. aureus* strains could be tested for the presence of mecA gene by PCR.

We thank he reviewer for the useful suggestion that gave us also the possibility to add more citations and better interpret the results. We agree with the reviewer that that it is not unreasonable to assume that isolates in our study were maybe indeed producers of penicillinase instead of being MRSA. We have now updated the Discussion (lines 412-420, see also below) to reflect this concept.

Discussion – lines 412-420: “None of the *S. aureus* isolates, except one, were found resistant to cefoxitin or oxacillin, despite being resistant to penicillin, suggesting that penicillin-resistant *S. aureus* isolates in this study were maybe indeed producers of penicillinase instead of being MRSA. This might be related to the fact that since the first report of *S. aureus* resistant to methicillin detected in a dairy herd in the United Kingdom (Garcia-Alvarez *et al*., 2011) and from the first isolation in 2012, of both *mecA* and *mecC* LA-MRSA in bulk milk from dairy cattle in the UK (Paterson *et al*., 2012), frequency of detection of *mecA* and *mecC* LA-MRSA in the UK, gathered from surveillance and large-scale dairy cattle studies, ([Paterson *et al*., 2012](https://www.ncbi.nlm.nih.gov/pmc/articles/PMC6751287/#B41), [2014](https://www.ncbi.nlm.nih.gov/pmc/articles/PMC6751287/#B42)) remained low (Anjum *et al*., 2019). The low frequency of resistance to cefoxitin or oxacillin found in our cohort is possibly reflecting that LA-MRSA was present in the UK, possibly at a low prevalence level.”

Anjum, M. F., Marco-Jimenez, F., Duncan, D., Marín, C., Smith, R. P., & Evans, S. J. (2019). Livestock-Associated Methicillin-Resistant Staphylococcus aureus From Animals and Animal Products in the UK. Frontiers in microbiology, 10, 2136. <https://doi.org/10.3389/fmicb.2019.02136>

García-Álvarez L, Holden MT, Lindsay H, Webb CR, Brown DF, Curran MD, Walpole E, Brooks K, Pickard DJ, Teale C, Parkhill J, Bentley SD, Edwards GF, Girvan EK, Kearns AM, Pichon B, Hill RL, Larsen AR, Skov RL, Peacock SJ, Maskell DJ, Holmes MA. Meticillin-resistant Staphylococcus aureus with a novel mecA homologue in human and bovine populations in the UK and Denmark: a descriptive study. Lancet Infect Dis. 2011 Aug;11(8):595-603. doi: 10.1016/S1473-3099(11)70126-8

Paterson, G. K., Larsen, J., Harrison, E. M., Larsen, A. R., Morgan, F. J., Peacock, S. J., Parkhill, J., Zadoks, R. N., & Holmes, M. A. (2012). First detection of livestock-associated meticillin-resistant Staphylococcus aureus CC398 in bulk tank milk in the United Kingdom, January to July 2012. Euro surveillance : bulletin Europeen sur les maladies transmissibles = European communicable disease bulletin, 17(50), 20337.

Paterson, G. K., Harrison, E. M., & Holmes, M. A. (2014). The emergence of mecC methicillin-resistant Staphylococcus aureus. Trends in microbiology, 22(1), 42–47. https://doi.org/10.1016/j.tim.2013.11.003

- Please revise titles of all tables. Tables and figures should be self-explanatory, thus all information required should be in the titles and legends.

We have edited the titles and legends of all tables (see also below for the titles). Table 1 on the original manuscript was removed and replaced by the new Figure 2 in the revised manuscript. The antimicrobial susceptibility profile of an isolate was miswritten in the original table 1 and on the manuscript, although the analysis was performed correctly. This was edited in the revised version. For clarity, we converted Table 1 into a plot (Figure 2). Therefore, Tables 2, 3 and 4 were renamed to Tables 1, 2 and 3 respectively.

Figure 2: “UpSet plot comparing the profiles of benzylpenicillin-resistant *Staphylococcus aureus* isolates. The total size of resistant *S. aureus* isolates is shown on the left bar plot. Antibiotic-resistant profiles of *S. aureus* isolates are visualized by the bottom plot and the occurrence is represented on the top bar plot.”

Table 1: “Statistical evaluation of the 4 peaks with an overall frequency of appearance higher than 30% based on the multidrug resistant vs susceptible data set. “

Table 2: “Statistical evaluation of the 5 peaks with an overall frequency of appearance higher than 30% based on the benzylpenicillin resistant only vs susceptible data set. “

Table 3. Annotation of the S. aureus proteins corresponding to the five MALDI-TOF peaks recognized as significant by the trained classifiers: peak mass charge ratio, predicted protein mass, top PSI-BLAST matches, conserved domain analyses, cellular locations and overexpressed classes are shown.

- Why the authors didn't carry out the analysis of penicillin-resistant versus multidrug-resistant isolates? This would help clarifying the role of the determined biomarkers in each resistance profile.

The classifiers could perform a good classification because the benzylpenicillin resistant and MDR/XDR isolates were theoretically both very different from the susceptible isolates. In general, the greater the differences, the more likely a good separation will be achieved. The 15 MDR isolates (13 MDR and 2XDR), are all resistant to benzylpenicillin. Therefore, there is a bias towards peaks determining resistance or susceptibility to benzylpenicillin, which may explain why all 4 multidrug discriminant peaks occurred within the set of benzylpenicillin-only discriminant peaks. Therefore, comparing the MDR/XDR against the benzylpenicillin resistant class might lead to a benzylpenicillin driven result. This is why we have not compared one versus the other

- The sentence starting at line 256 should be revised as it is too long.

The sentence starting at line 256 was corrected. See Results – lines 336-344 and below

Results - Lines 336-344: “GO and KEGG analyses of the network showed enrichment for ribosome, nucleic acid binding and catalytic activity (Figure 7).

Tetracycline resistance protein (TetM) and elongation factor G (FusA) were found as first neighbours of RpmJ and RpmD based on the experimental findings of their homologs in E. coli [28, 29]. Four additional proteins (MecA, BlaZ, PbpA and metallo-beta-lactamase (MBL)) were associated with beta-lactams, rRNA adenine N-6-methyltransferase (ErmA), macrolides resistance, multidrug efflux pump (NorA) and ABC transporter protein (ABC-2). These proteins were found to interact with some first neighbours of the discriminant proteins in the network.”

- I suggest revising the names of the proteins included in the text. Usually we use italics when referring to the name of the gene, while the name of the protein coded by the gene starts with capital letter and it is in regular font.

We have corrected them throughout the manuscript

- I suggest including one more information field in Table 4: for each biomarker/protein include the group of isolates (susceptible, penicillin-resistant or multidrug-resistant) in which the peptide seems to be over expressed.

We have now included a new column was included in Table 3 (named Table 4 on the original manuscript) to include the group of isolates (susceptible, penicillin-resistant or multidrug-resistant) in which the peptide seems to be over expressed

- There is no explanation of what the abbreviation "ABR" in line 301 means.

We have edited the text to “antibiotic resistance” on Discussion Line 392

- Please revise the paragraph starting at page 14 as it is one-page long.

We have split the paragraph on the original manuscript at page 14 into smaller paragraphs.

Discussion lines 426-455: “The notion that components of the ribosome are important in the growth rate and antibiotic resistance of bacteria is a well-known concept [56]. Among those determinants involved in intrinsic resistance, ribosomal proteins have been found to deal with the general response to stress [57]. Similarly, recent findings highlighted the existence of ribosomal mutations conferring resistance to antibiotics of several classes not targeting the ribosome [56]. Specifically, it has been shown that ribosomal mutations can contribute to the evolution of multidrug-resistant profiles, by inducing ribosomal mis-assembly, that in turn leads to a systematic transcriptional cell alteration, ultimately impacting resistance to multiple antibiotics by interfering with different cellular pathways [56]. *RpmJ* was shown to be up-regulated in *Pseudomonas aeruginosa* when treated with ciprofloxacin and fluoroquinolone [58] and similarly in *S. epidermidis* [59]. Moreover, *rpmJ* was shown to confer intrinsic multidrug resistance to a varied set of antibiotics (nitrofurantoin, sulfamethoxazole, rifampicin, tetracycline, vancomycin, ampicillin, colistin, erythromycin) in *E. coli*, where deletion of this gene caused the bacteria to become more sensitive than wild type [60]. In comparison, fewer literature works have been published about *rpmD* and antibiotic resistance. Sharma-Kuinekel and collaborators showed that *rpmD* was downregulated in *S. aureus* strains which had the antibiotic tolerance related LytSR system silenced [61].

The discriminant protein DNA-binding HU protein was found essential in the bacterial survival and growth of *S. aureus* [62]. It was also previously found to be correlated to antibiotic resistance by being upregulated in the mutant *S. aureus* isolates with silenced serine/threonine kinase PknB, which also has a penicillin-binding domain [63]. Besides the proteins with known functions, we also identified two hypothetical proteins, but we were unable to find any evidence so far linking them to antibiotic resistance. Although it was not possible for us to identify the function of these hypothetical proteins, by applying PSI-BLAST and PSORTb v3.0 together with 3D threading modelling searches, the hypothetical proteins are predicted to be involved in pathways such as antibiotic metabolic process, lipid/protein transport and ion binding.”

- I don't think there is enough data supporting the hypothesis raised in lines 353-355.

As correctly pointed out by the reviewer more data is needed to support the hypothesis. We have now deleted the sentence.

- As there were included in the study isolates obtained from a same animal, it would be interesting to know if these isolates coming from the same animal also presented the same antimicrobial susceptibility profile, as it would maybe mean that they represent the same strain.

We have generated a new supplementary material Supplementary Table 2, where for the isolates coming from the same animals, we detailed the antimicrobial susceptibility profile for each antimicrobial tested in the study. For simplicity we named the animals with alphabetical letters, so it is easier for the reader to understand the association between the animals and the susceptibility profiles. As correctly pointed out by the Reviewer, we found several isolates coming from the same with identical susceptibility profiles. We have summarized them below. However, for full details please check Supplementary Table 2. We have also updated the Results and Discussion (Results – 167-168 and Discussion 410-412 and see below) to reflect the reviewer comment

We have also summarised this information below for the Reviewer.

1. For animal A, isolates 5259 and 7841 had the same antimicrobial susceptibility profile and same MIC values for each one of the tested antimicrobials;
2. For animal B, isolates 6118, 6125 and 6647 had the same antimicrobial susceptibility profile but different MIC values for each one of the tested antimicrobials;
3. For animal C, isolates 3788 and 7100 had the same antimicrobial susceptibility profile but different MIC values for each one of the tested antimicrobials;
4. For animal D isolates 3773 and 3790 had the same antimicrobial susceptibility profile and same MIC values for each one of the tested antimicrobials;
5. For animal E, isolates 3765, 3767 and 3785 had the same antimicrobial susceptibility profile for some of the tested antimicrobials, but different antimicrobial susceptibility profiles for four of the tested antimicrobials;
6. For animal F, isolates 1945, 3758 and 3763 had the same antimicrobial susceptibility profile but different MIC values for each one of the tested antimicrobials.

Results lines: 167-168: “Supplementary Table 2 indicates the antimicrobial susceptibility profile of the resistant isolates that were obtained from the same animal.

Discussion- lines 410-412: “Several isolates were obtained from the same animal, some of them also presented the same antimicrobial susceptibility profile, possibly suggesting that they represent the same strain.”

- Regarding generation of MALDI spectra, I suggest including in the Methodology which equipment was used, which matrix was used and which was the original *m/z* range acquired. In addition, why only 6 replicates were obtained for each isolate? In there any reference that supports this? Usually in the literature, and according to Bruker, at least 20 replicates are required for tests like this.

This information was missing indeed. We have now edited the Methods providing more information (See methods line 621-635)

Methods – lines 621-635: The pure cultures were then analysed using the Time-of-flight (TOF) MALDI mass spectrometer (Bruker Daltonics, Billerica, MA), Microflex – Flex Control Version 3.4, Bruker Daltonics. The order of sample and technical replicate analysis was randomised, the Bruker Bacterial Test Standard (BTS) (Bruker Daltonics) was used for calibration control on every plate. For each isolate, six technical replicates were generated from 240 desorption’s per replicate (6 x 40 shots), and protein mass spectra acquired in the range 2000 to 20,000 Da were generated. Spectra were compared visually using Biotyper 3.1 (Bruker Daltonics) to remove low intensity spectra or spectra with substantial background noise. All the samples used in this study were further analysed visually on Matlab for insufficient resolution (defined as a measure to distinguish two peaks of slightly different *m/z* values (Mulvaney, 1993)), low intensity or substantial background. However, no samples were discarded for these reasons. Technical replicates were further compared using composite correlation indices (CCI) to remove dissimilar spectra with CCI < 0.99 (Archer *et al*., 2017). At least three good quality spectra per isolate were required for inclusion of the isolate in the analysis. Moreover, when three qualifying technical replicates could not be obtained the sample was re-analysed in order to get at least 3 replicates. All the 82 isolates used in this study had three good quality technical replicates.

Archer SC, Bradley AJ, Cooper S, Davies PL, Green MJ. Prediction of Streptococcus uberis clinical mastitis risk using Matrix-assisted laser desorption ionization time of flight mass spectrometry (MALDI-TOF MS) in dairy herds. Prev Vet Med. 2017 Sep 1;144:1-6. doi: 10.1016/j.prevetmed.2017.05.015. Epub 2017 May 17. PMID: 28716189; PMCID: PMC5529212.

R.L. Mulvaney (1993), Mass Spectrometry, In R. Knowles (Ed.), T. H. Blackburn (Ed.), Nitrogen Isotope Techniques, Academic Press, pp. 11-57, ISBN 9780124169654, https://doi.org/10.1016/B978-0-08-092407-6.50007-9.

- Throughout the manuscript, revise the term "*S. aureus*" as it should always be italics.

Corrected

**Additional edits to the manuscript**

A note for the Reviewers and the Editor, during the revision we realized that in the original submission, we have erroneously labelled a hypothetical protein in the S. aureus (strain NCTC 8325/PS 47) proteome as HP1 (one of our discriminant proteins). The mislabelling was caused by the fact that HP1 has not been annotated yet in the S. aureus (strain NCTC 8325/PS 47) proteome and we mistakenly selected a false positive protein (not significant e-value in Blast). However, HP1 is present in the model bovine mastitis S. aureus (strain Newbould 305 (ATCC 29740) proteome, that was the proteome used by us to find correspondences between individual peaks selected by the machine learning-based classifiers and actual proteins of S. aureus. Hence, the peak m/z 4807.21 was correctly associate to HP1 but could not be modelled it in the S. aureus (strain NCTC 8325/PS 47) protein-protein interactions network (PPI). We have now rerun the PPI analysis using the same settings as in the original submission but with four discriminant proteins instead of five (without HP1). All the interactions scores (the average number of neighbours, clustering coefficient, network density and network heterogeneity) were recalculated. The new PPI scores are very similar to the original ones. In particular, 153 proteins were modelled in the PPI instead of the original 159 proteins. A total of 149 first neighbour proteins were found instead of the original 154. A connectivity clustering coefficient of 0.728 was calculated with respect to the original 0.719. The average number of neighbours per protein was 68.719 instead of the original 65.875. In terms of network density, the value found was 0.452 (the original value was 0.414) and for the network heterogeneity the predicted value was 0.528 (the original value was 0.576). All the values calculated for the complete S. aureus proteome network remained the same. The antibiotic-resistant proteins found with ResFinder amongst the first shell interacting partners remained the same even without HP1. We apologise for this mistake, however we believe that this was a minor change as the only part of the work affected by it is the PPI, that although ran without HP1 performed very similarly. We have updated the text on the results lines 368-375 and 334-335, Methods 811-812, and we have generated a new high-quality Figure 6 showing the PPI, see also below:

Results - lines 368-375: “Notably, the PPI analysis of the benzylpenicillin-resistant proteome, 153 proteins – a total of 4 discriminant proteins and 149 first neighbour proteins – showed higher connectivity (clustering coefficient 0.728) than the complete *S. aureus* proteome network (clustering coefficient 0.421). The average number of neighbours per protein was 68.719 in the benzylpenicillin-resistant proteome network and 27.190 in the complete *S. aureus* proteome network. In terms of network density, the values ranged between 0.452 (benzylpenicillin-resistant proteome network) and 0.009 (complete *S. aureus* proteome network) and for the network heterogeneity the values ranged between 0.528 benzylpenicillin-resistant proteome network) and 1.243 (complete *S. aureus* proteome network).”

Results lines 334-335: “It should be noted that HP1 could not be found in the *S. aureus* (strain NCTC 8325/PS 47) proteome that was available in STRING database.”

Methods lines 741-742: “The PPI dataset of *S. aureus* (strain NCTC 8325/PS 47) was obtained from the STRING database”

Figure 6 legend: “Protein-protein interaction network of the proteins found to correspond to the MALDI-TOF spectral peaks recognized as discriminant between benzylpenicillin-resistant and susceptible isolates. The four discriminant proteins (RpmJ, RpmD, HU and hypothetical protein 2 (HP2) are shown as green circles and their first neighbour interactors are shown as orange circles. Amongst the first shell interacting partners, purple nodes represent the antibiotic-resistant proteins (BlaZ, NorA, MecA, PbpA, ErmA, ABC-2, TetM, FusA and MBL) predicted by ResFinder v3.1 (Zankari *et al.,* 2012).”

Zankari E, Hasman H, Cosentino S, Vestergaard M, Rasmussen S, Lund O, et al. Identification of acquired antimicrobial resistance genes. J Antimicrob Chemother. 2012;67(11):2640-4. Epub 2012/07/12. doi: 10.1093/jac/dks261. PubMed PMID: 22782487; PubMed Central PMCID: PMCPMC3468078.

Moreover, at Introduction lines 87-91 we updated the text by adding a more up to date reference and edit the text accordingly.

Introduction lines 87-91: “In 2000, Gentillini *et al*. (2000) indicated that beta-lactams (penicillins and cephalosporins), aminoglycosides, macrolides and lincosamides were the most commonly used antibiotics for treatment of bovine mastitis. In addition, according to a recent survey (Higham *et al.,* 2018) in 2018, penicillins, aminoglycosides and third/fourth generation cephalosporins were the most common antibiotics used on the treatment for bovine mastitis in the UK.”

Gentilini E, Denamiel G, Llorente P, Godaly S, Rebuelto M, DeGregorio O. Antimicrobial susceptibility of Staphylococcus aureus isolated from bovine mastitis in Argentina. Journal of dairy science. 2000;83(6):1224-7.

Higham LE, Deakin A, Tivey E, Porteus V, Ridgway S, Rayner AC. A survey of dairy cow farmers in the United Kingdom: knowledge, attitudes and practices surrounding antimicrobial use and resistance. Veterinary Record. 2018;183(24):746.
