# Supplementary material for: Mass spectrometry and machine learning for the accurate diagnosis of benzylpenicillin and multidrug resistance of Staphylococcus aureus in bovine mastitis
Source: PLoS Comput Biol. 2021 Jun 11;17(6):e1009108. doi: 10.1371/journal.pcbi.1009108 (PMC8221797; doi:10.1371/journal.pcbi.1009108)
Supplement: Peer review history

# Peer review: Decision Letter 0

**DOI:** 10.1371/journal.pcbi.1009108.r001

**Authors:** Alice Carolyn McHardy, Mark Holmes

14 Jan 2021

Dear Dr Dottorini,

Thank you very much for submitting your manuscript "Mass spectrometry and machine learning for the accurate diagnosis of benzylpenicillin and multi-drug resistance of Staphylococcus aureus in bovine mastitis" for consideration at PLOS Computational Biology.

As with all papers reviewed by the journal, your manuscript was reviewed by members of the editorial board and by several independent reviewers. In light of the reviews (below this email), we would like to invite the resubmission of a significantly-revised version that takes into account the reviewers' comments.

Many apologies for the delay in getting these reviewers comments to you.

Although the reviewers both indicate that major revisions are required I do think that this is an interesting study and worthy of publication. I would encourage the authors to address their comments and edit the manuscript accordingly. There may be some comments that you disagree with and so please address these in your response to reviewers. The edited manuscript will be sent out to the same reviewers.

Thank you for your patience.

We cannot make any decision about publication until we have seen the revised manuscript and your response to the reviewers' comments. Your revised manuscript is also likely to be sent to reviewers for further evaluation.

When you are ready to resubmit, please upload the following:

[1] A letter containing a detailed list of your responses to the review comments and a description of the changes you have made in the manuscript. Please note while forming your response, if your article is accepted, you may have the opportunity to make the peer review history publicly available. The record will include editor decision letters (with reviews) and your responses to reviewer comments. If eligible, we will contact you to opt in or out.

[2] Two versions of the revised manuscript: one with either highlights or tracked changes denoting where the text has been changed; the other a clean version (uploaded as the manuscript file).

Important additional instructions are given below your reviewer comments.

Please prepare and submit your revised manuscript within 60 days. If you anticipate any delay, please let us know the expected resubmission date by replying to this email. Please note that revised manuscripts received after the 60-day due date may require evaluation and peer review similar to newly submitted manuscripts.

Thank you again for your submission. We hope that our editorial process has been constructive so far, and we welcome your feedback at any time. Please don't hesitate to contact us if you have any questions or comments.

Sincerely,

Mark Holmes

Guest Editor

PLOS Computational Biology

Alice McHardy

Deputy Editor

PLOS Computational Biology

***********************

Many apologies for the delay in getting these reviewers comments to you.

Although the reviewers both indicate that major revisions are required I do think that this is an interesting study and worthy of publication. I would encourage the authors to address their comments and edit the manuscript accordingly. There may be some comments that you disagree with and so please address these in your response to reviewers. The edited manuscript will be sent out to the same reviewers.

Thank you for your patience.

Reviewer's Responses to Questions

**Comments to the Authors:**

**Please note here if the review is uploaded as an attachment.**

Reviewer #1: The manuscript by Necati Esener and colleagues reports on the use of MALDI-ToF to detect antibiotic resistance profiles for benzylpenicillin and MDR S. aureus without prior exposure to antibiotics. Although this use of MALDI-ToF has been reported before, this is the first study to my understanding that has used S. aureus isolates from bovine mastitis cases. The manuscript is interesting and has the potential to rapidly speed up the appropriate treatment for bovine mastitis. However, I have several major concerns regarding the manuscript that I believe should be addressed before it is suitable for publication. I have divided these concerns by reference to manuscript sections.

Introduction:

• This type of study has been completed previously on S. aureus isolates, albeit not one from bovine mastitis cases. This should be addressed in the introduction in more detail.

Methodology:

• There is little information on isolation of S. aureus isolates. What media was used, growth conditions etc? Were they stored in ultra-low temperature freezers and for how long? Did this differ between collection groups or resistance groups?

• There is too little information on MALDI-ToF acquisition. What instrument was used? I imagine that it was a BioTyper but generic reference to MALDI-ToF is misleading. Were samples randomised during running, including randomisation of technical repeats? What was used as a calibrant control? Although there is reference to a previous publication, in my opinion this information should be fully reported in the manuscript.

• The authors states that technical replicates with an insufficient resolution were removed. What were the criteria for this? Was this random or related to sample source etc?

• 82 isolates were started with and 82 were used in data processing. The authors refer in line 419-420 that isolates with less than three technical replicates were removed. Did this happen?

• Spectral peak selection: authors refer in line 450 that peaks selected if present in at least 30% of all spectra. Is this 30% of samples or 30% of all generated spectra? Why was a cut-off chosen – could this remove peaks that differentiated between susceptible/resistant isolates as the group sizes were not balanced?

• There is a potential issue in that the data pre-processing was completed on all isolates in one. This may bias the resulting mass spectra and lead to reduced reproducibility of the classification model when used on further validation isolates. Ideally, the authors should complete data pre-processing and statistical mode creation, in my opinion, on a 2/3 training set and then the entire pipeline is validated against the remaining 1/3 of isolates.

• Data availability: the spectra should really be deposited in a public repository according to PLoS Computational Biology guidelines.

Results:

• If cross-validation was performed, then the confidence intervals of the classification models should be reported specifically. Although this is given in Figures 2 and 3, exact values should be written somewhere.

• There is always a concern with this type of analysis in that they are picking up strain differences rather than susceptibility/resistance differences. Were the isolates strain typed? As two of the four differential proteins were of ribosomal origins, this may be a possibility and an important consideration for the authors to address.

• It was also be good to show a breakdown of isolates by susceptibility/resistance by farm location. Were they evenly distributed amongst farms or was the model driven by differences in location of collection?

• Was there any correlation between MIC levels and protein intensities?

• As for earlier comment, this is not the first use of MALDI-ToF for in antibiotic susceptibility profiling in S. aureus. Did previous studies, albeit using S. aureus isolated from humans or non-bovine sources show the same proteins as differential?

Reviewer #2: The work aims to develop a MALDI-TOF-based approach to discriminate between antibiotic-resistant and -susceptible S. aureus isolates recovered from bovine mastitis. Since this approach is faster and simpler than conventional ways for determining antimicrobial susceptibility, it represents a potential alternative that could be useful in the veterinary field. However, few points should be addressed throughout the manuscript, as follows:

- In the Introduction, the authors mention the emergence of penicillinase-producing S. aureus strains back in the 50's. I believe it should be also mentioned in the Introduction that production of alternative PBPs (coded by mecA for example) are nowadays more important than penicllinase production.

- Throughout the manuscript revise the term "multidrug-resistance" for standardization purposes, as in certain places it is written "multi-drug resistance" (including in the title of the manuscript).

- Were the 82 S. aureus isolates of the study chosen among a larger collection of isolates? If so, which were the selection criteria? Also, wouldn't 82 isolates be a small number of samples to be used as a test collection for a new methodology?

- Usually, classification of bacteria as multidrug resistant requires that they show resistance to at least three different classes of antibiotics. That is quite a common sense in the literature. Why in the study the authors considered multidrug resistance when isolates were resistant to two antibiotics? Is there any reference that supports this?

- One result stands out: none of the S. aureus isolates was resistant tp cefoxitin or oxacillin, despite being resistant to penicillin. For human isolates of S. aureus, initial screening and detection of MRSA is usually based on the in vitro susceptibility to cefoxitin or oxacillin. In this regard, one could suggest that penicillin-resistant S. aureus isolates in this study were maybe indeed producers of penicillinase instead of being MRSA. This could be included in the discussion, or S. aureus strains could be tested for the presence of mecA gene by PCR.

- Please revise titles of all tables. Tables and figures should be self-explanatory, thus all information required should be in the titles and legends.

- Why the authors didn't carry out the analysis of penicillin-resistant versus multidrug-resistant isolates? This would help clarifying the role of the determined biomarkers in each resistance profile.

- The sentence starting at line 256 should be revised as it is too long.

- I suggest revising the names of the proteins included in the text. Usually we use italics when referring to the name of the gene, while the name of the protein coded by the gene starts with capital letter and it is in regular font.

- I suggest including one more information field in Table 4: for each biomarker/protein include the group of isolates (susceptible, penicillin-resistant or multidrug-resistant) in which the peptide seems to be over expressed.

- There is no explanation of what the abbreviation "ABR" in line 301 means.

- Please revise the paragraph starting at page 14 as it is one-page long.

- I don't think there is enough data supporting the hypothesis raised in lines 353-355.

- As there were included in the study isolates obtained from a same animal, it would be interesting to know if these isolates coming from the same animal also presented the same antimicrobial susceptibility profile, as it would maybe mean that they represent the same strain.

- Regarding generation of MALDI spectra, I suggest including in the Methodology which equipment was used, which matrix was used and which was the original m/z range acquired. In addition, why only 6 replicates were obtained for each isolate? In there any reference that supports this? Usually in the literature, and according to Bruker, at least 20 replicates are required for tests like this.

- Throughout the manuscript, revise the term "S. aureus" as it should always be italics.

**********

**Have all data underlying the figures and results presented in the manuscript been provided?**

Large-scale datasets should be made available via a public repository as described in the *PLOS Computational Biology* data availability policy, and numerical data that underlies graphs or summary statistics should be provided in spreadsheet form as supporting information.

Reviewer #1: **No: **The authors state that "Yes - all data are fully available without restriction" and that this information can be requested from Prof. Andrew Bradley. However, in the manuscript it says that it remains the property of QMMS Ltd. I see no reason why it cannot be deposited in a public repository.

Reviewer #2: Yes

**********

PLOS authors have the option to publish the peer review history of their article (what does this mean?). If published, this will include your full peer review and any attached files.

If you choose “no”, your identity will remain anonymous but your review may still be made public.

**Do you want your identity to be public for this peer review?** For information about this choice, including consent withdrawal, please see our Privacy Policy.

Reviewer #1: No

Reviewer #2: No

Figure Files:

While revising your submission, please upload your figure files to the Preflight Analysis and Conversion Engine (PACE) digital diagnostic tool, https://pacev2.apexcovantage.com. PACE helps ensure that figures meet PLOS requirements. To use PACE, you must first register as a user. Then, login and navigate to the UPLOAD tab, where you will find detailed instructions on how to use the tool. If you encounter any issues or have any questions when using PACE, please email us at figures@plos.org.

Data Requirements:

Please note that, as a condition of publication, PLOS' data policy requires that you make available all data used to draw the conclusions outlined in your manuscript. Data must be deposited in an appropriate repository, included within the body of the manuscript, or uploaded as supporting information. This includes all numerical values that were used to generate graphs, histograms etc.. For an example in PLOS Biology see here: http://www.plosbiology.org/article/info%3Adoi%2F10.1371%2Fjournal.pbio.1001908#s5.

Reproducibility:

To enhance the reproducibility of your results, PLOS recommends that you deposit laboratory protocols in protocols.io, where a protocol can be assigned its own identifier (DOI) such that it can be cited independently in the future. For instructions, please see http://journals.plos.org/compbiol/s/submission-guidelines#loc-materials-and-methods

# Author comment: Author response to Decision Letter 0

**DOI:** 10.1371/journal.pcbi.1009108.r002

25 Mar 2021

Attachment Submitted filename: Response to Reviewers.docx Click here for additional data file.

# Peer review: Decision Letter 1

**DOI:** 10.1371/journal.pcbi.1009108.r003

**Authors:** Alice Carolyn McHardy, Mark Holmes

22 May 2021

Dear Dr Dottorini,

We are pleased to inform you that your manuscript 'Mass spectrometry and machine learning for the accurate diagnosis of benzylpenicillin and multidrug resistance of Staphylococcus aureus in bovine mastitis' has been provisionally accepted for publication in PLOS Computational Biology.

Before your manuscript can be formally accepted you will need to complete some formatting changes, which you will receive in a follow up email. A member of our team will be in touch with a set of requests.

Please note that your manuscript will not be scheduled for publication until you have made the required changes, so a swift response is appreciated.

IMPORTANT: The editorial review process is now complete. PLOS will only permit corrections to spelling, formatting or significant scientific errors from this point onwards. Requests for major changes, or any which affect the scientific understanding of your work, will cause delays to the publication date of your manuscript.

Should you, your institution's press office or the journal office choose to press release your paper, you will automatically be opted out of early publication. We ask that you notify us now if you or your institution is planning to press release the article. All press must be co-ordinated with PLOS.

Thank you again for supporting Open Access publishing; we are looking forward to publishing your work in PLOS Computational Biology. 

Best regards,

Mark Holmes

Guest Editor

PLOS Computational Biology

Alice McHardy

Deputy Editor

PLOS Computational Biology

***********************************************************

Thank you for your resubmission. The reviewers are now happy with your manuscript and I'm happy to accept it for publication.

Please accept my apologies again for the delays you experienced in the review of this paper.

Reviewer's Responses to Questions

**Comments to the Authors:**

**Please note here if the review is uploaded as an attachment.**

Reviewer #1: The authors have provided a comprehensive response and modifications to their manuscript in their revised submission and I am satisfied that all of my concerns have been addressed. I would be happy to support this manuscript for publication.

Reviewer #2: The authors have fully and properly addressed all initial major concerns, and the revised version of the manuscript is now suitable for publication.

**********

**Have the authors made all data and (if applicable) computational code underlying the findings in their manuscript fully available?**

The PLOS Data policy requires authors to make all data and code underlying the findings described in their manuscript fully available without restriction, with rare exception (please refer to the Data Availability Statement in the manuscript PDF file). The data and code should be provided as part of the manuscript or its supporting information, or deposited to a public repository. For example, in addition to summary statistics, the data points behind means, medians and variance measures should be available. If there are restrictions on publicly sharing data or code —e.g. participant privacy or use of data from a third party—those must be specified.

Reviewer #1: **No: **

Reviewer #2: None

**********

PLOS authors have the option to publish the peer review history of their article (what does this mean?). If published, this will include your full peer review and any attached files.

If you choose “no”, your identity will remain anonymous but your review may still be made public.

**Do you want your identity to be public for this peer review?** For information about this choice, including consent withdrawal, please see our Privacy Policy.

Reviewer #1: No

Reviewer #2: **Yes: **Tatiana Pinto

# Editor report: Acceptance letter

**DOI:** 10.1371/journal.pcbi.1009108.r004

**Authors:** Alice Carolyn McHardy, Mark Holmes

8 Jun 2021

PCOMPBIOL-D-20-00864R1 

Mass spectrometry and machine learning for the accurate diagnosis of benzylpenicillin and multidrug resistance of Staphylococcus aureus in bovine mastitis

Dear Dr Dottorini,

I am pleased to inform you that your manuscript has been formally accepted for publication in PLOS Computational Biology. Your manuscript is now with our production department and you will be notified of the publication date in due course.

The corresponding author will soon be receiving a typeset proof for review, to ensure errors have not been introduced during production. Please review the PDF proof of your manuscript carefully, as this is the last chance to correct any errors. Please note that major changes, or those which affect the scientific understanding of the work, will likely cause delays to the publication date of your manuscript. 

Soon after your final files are uploaded, unless you have opted out, the early version of your manuscript will be published online. The date of the early version will be your article's publication date. The final article will be published to the same URL, and all versions of the paper will be accessible to readers.

Thank you again for supporting PLOS Computational Biology and open-access publishing. We are looking forward to publishing your work! 

With kind regards,

Katalin Szabo

PLOS Computational Biology | Carlyle House, Carlyle Road, Cambridge CB4 3DN | United Kingdom ploscompbiol@plos.org | Phone +44 (0) 1223-442824 | ploscompbiol.org | @PLOSCompBiol
